# Mechanisms underlying the role of endoplasmic reticulum stress in the placental injury and fetal growth restriction in an ovine gestation model

**DOI:** 10.1186/s40104-023-00919-z

**Published:** 2023-09-11

**Authors:** Hao Zhang, Xia Zha, Yi Zheng, Xiaoyun Liu, Mabrouk Elsabagh, Hongrong Wang, Honghua Jiang, Mengzhi Wang

**Affiliations:** 1 Laboratory of Metabolic Manipulation of Herbivorous Animal Nutrition, College of Animal Science and Technology, Yangzhou University, Yangzhou, 225009 P. R. China; 2 Joint International Research Laboratory of Agriculture and Agri-Product Safety, Ministry of Education of China, Yangzhou University, Yangzhou, 225009 P. R. China; 3 Department of Animal Production and Technology, Faculty of Agricultural Sciences and Technologies, Niğde Ömer Halisdemir University, Nigde, 51240 Turkey; 4 Department of Nutrition and Clinical Nutrition, Faculty of Veterinary Medicine, Kafrelsheikh University, KafrelSheikh, Egypt; 5 Department of Pediatrics, Northern Jiangsu People’s Hospital, Clinical Medical College, Yangzhou University, Yangzhou, 225001 China; 6 State Key Laboratory of Sheep Genetic Improvement and Healthy Production, Xinjiang Academy of Agricultural Reclamation Science, Shihezi, 832000 China

**Keywords:** Autophagy, Bisphenol A, Endoplasmic reticulum stre*s*s, Fetal growth restriction, Inflammatory responses, Sheep

## Abstract

**Background:**

Exposure to bisphenol A (BPA), an environmental pollutant known for its endocrine-disrupting properties, during gestation has been reported to increase the risk of fetal growth restriction (FGR) in an ovine model of pregnancy. We hypothesized that the FGR results from the BPA-induced insufficiency and barrier dysfunction of the placenta, oxidative stress, inflammatory responses, autophagy and endoplasmic reticulum stress (ERS). However, precise mechanisms underlying the BPA-induced placental dysfunction, and subsequently, FGR, as well as the potential involvement of placental ERS in these complications, remain to be investigated.

**Methods:**

In vivo experiment, 16 twin-pregnant (from d 40 to 130 of gestation) Hu ewes were randomly distributed into two groups (8 ewes each). One group served as a control and received corn oil once a day, whereas the other group received BPA (5 mg/kg/d as a subcutaneous injection). In vitro study, ovine trophoblast cells (OTCs) were exposed to 4 treatments, 6 replicates each. The OTCs were treated with 400 μmol/L BPA, 400 μmol/L BPA + 0.5 μg/mL tunicamycin (Tm; ERS activator), 400 μmol/L BPA + 1 μmol/L 4-phenyl butyric acid (4-PBA; ERS antagonist) and DMEM/F12 complete medium (control), for 24 h.

**Results:**

In vivo experiments, pregnant Hu ewes receiving the BPA from 40 to 130 days of pregnancy experienced a decrease in placental efficiency, progesterone (P4) level and fetal weight, and an increase in placental estrogen (E2) level, together with barrier dysfunctions, OS, inflammatory responses, autophagy and ERS in type A cotyledons. In vitro experiment, the OTCs exposed to BPA for 24 h showed an increase in the E2 level and related protein and gene expressions of autophagy, ERS, pro-apoptosis and inflammatory response, and a decrease in the P4 level and the related protein and gene expressions of antioxidant, anti-apoptosis and barrier function. Moreover, treating the OTCs with Tm aggravated BPA-induced dysfunction of barrier and endocrine (the increased E2 level and decreased P4 level), OS, inflammatory responses, autophagy, and ERS. However, treating the OTCs with 4-PBA reversed the counteracted effects of Tm mentioned above.

**Conclusions:**

In general, the results reveal that BPA exposure can cause ERS in the ovine placenta and OTCs, and ERS induction might aggravate BPA-induced dysfunction of the placental barrier and endocrine, OS, inflammatory responses, and autophagy. These data offer novel mechanistic insights into whether ERS is involved in BPA-mediated placental dysfunction and fetal development.

**Supplementary Information:**

The online version contains supplementary material available at 10.1186/s40104-023-00919-z.

## Background

Bisphenol A (BPA), a common environmental pollutant known for its endocrine-disrupting properties, is used in various applications such as polycarbonate, epoxy resins for plastics, metal cans and food storage containers [1]. In 2007, approximately 2,500 tonnes of BPA were released into the environment according to the Toxics Release Inventory in the USA [2]. The detection of low levels of BPA in human bodily fluids like urine, blood and breast milk has raised concerns regarding its potential impact on public health and safety [3].

The placenta is a highly specialized organ that plays a crucial role in the healthy development and growth of the fetus during pregnancy [4]. Placental trophoblasts are a vital component of the placenta and play a fundamental role in ensuring a successful pregnancy. Placental trophoblast dysfunctions have been linked to various pregnancy disorders, including placenta accreta/increta/percreta, preeclampsia, spontaneous abortion, and fetal growth restriction (FGR) [5]. The syncytiotrophoblast cells are the outermost layer of the placenta and are responsible for nutrition and gaseous exchange. In species with a hemochorial type of placentas, where syncytiotrophoblast cells are exposed to maternal blood, there is a risk of exposure to toxic substances carried in the maternal circulation [6]. The placenta acts not only as a detoxifier but also as an endocrine organ making it susceptible to compounds that disrupt its endocrine functions [7]. Exposure to BPA has been reported to have detrimental impacts on the development of the mammary gland, reproductive functions, metabolic activity, and cognitive performance [8]. Moreover, BPA can pass from mother to offspring through the placenta [9], making it a significant environmental risk during pregnancy. Studies have indicated that BPA can cross the placental barrier, leading to developmental and functional problems in the placenta and posing a significant risk of FGR [10–12]. The FGR is not only a significant risk factor for fetal mortality, but also is associated with an increased likelihood of developing chronic non-communicable diseases in adulthood, such as cardiovascular disease, cancer, metabolic disorders, chronic immunological diseases, and neurological abnormalities [13]. Overwhelming data indicated that BPA was a major environmental stress factor triggering FGR. However, the exact mechanism by which BPA causes FGR remains unclear.

The endoplasmic reticulum (ER) is a membrane-bound organelle in eukaryotic cells with multiple functions including protein and lipid synthesis, detoxification of xenobiotics and storage of cellular calcium (Ca^2+^) [14]. Various factors, including pathological, physiological and biochemical stimuli, can disrupt the proper folding of the protein in ER leading to the accumulation of unfolded or misfolded proteins in the ER and triggering endoplasmic reticulum stress (ERS) and unfolded protein response (UPR) [15]. The UPR can be initiated in response to ERS during challenging physiological conditions. This response involves three proteins associated with the ER membrane: PKR-like eukaryotic initiation factor 2a kinase (PERK), activating transcription factor-6 (ATF6), and inositol requiring enzyme 1 (IRE-1) [16]. ERS has been linked to inflammation [17] and affects placental development and functions, fetal growth, progesterone secretion, and the expression of steroidogenic enzymes [18, 19]. The signalling of ERS and UPR impacts the functionally active proteins produced by placental cells, thereby playing a crucial role in the pathophysiological regulation of later stages of placental and fetal development, as well as parturition [20, 21]. However, there is limited data on the role of ERS in the placental barrier and endocrine dysfunction, oxidative stress (OS), inflammatory responses, and FGR induced by BPA in pregnant ewes.

Sheep are suitable models for studying placental function despite differences in placental structure between primates and ruminants [22]. This is because sheep trophoblast contains multinucleate cells similar to the syncytiotrophoblast cells in humans [23]. In addition, it has been shown that the endogenous retrovirus envelope gene, enJSRV, is essential for the fusogenic process that results in multinucleated cells in both sheep and humans [24]. To fully understand the effects of bisphenol compounds on placental function, researchers utilized a comparative approach using sheep as an animal model [22]. This model offered initial proof of the negative effects of bisphenol compounds on placental development and endocrine function in pregnant sheep [22]. In the current study, the mechanisms of BPA-triggered ERS, OS, and inflammatory responses were examined using the ovine placenta and primary cultured ovine trophoblast cells (OTCs). We also used pharmacological intervention to examine the role of ERS in the placental barrier and endocrine dysregulation, OS, and inflammatory responses induced by BPA.

## Methods

All study protocols were approved by the institutional animal care and use committee of Yangzhou University (Ethical approval number: SYXK 2016–0019).

### Animals

The study was conducted at the Jiangyan Experimental Station in Taizhou, Jiangsu Province, China. Sheep indoor pens were equipped with heating radiators to sustain an average temperature of 15 ± 0.72 °C while illumination was controlled automatically to simulate the natural surroundings photoperiod during the whole experiment [25]. Thirty-two multiparous Hu ewes of similar BW (40.5 ± 1.6 kg), age (18.3 ± 0.6 months) and BCS (2.54 ± 0.17; scale 0 = emaciation to 5 = obesity) [26] were treated with the antiparasitic ivermectin (0.2 mg/kg/BW) and then were synchronized for estrus for 12 d using the intravaginal progesterone sponges (30 mg; Pharmp PTY, Herston City, Australia). The estrus behaviour was observed twice a day (at 0800 and 1600 h) using 3 teaser rams one day after removing sponges. The ewes were artificially inseminated with fresh semen after two days of sponges removal (d 0 of gestation) and then were individually housed in separate pens (1.20 m × 1.80 m) until d 40 of gestation. After 40 days of gestation, 16 ewes were subjected to scanning by ultrasonography (Asonics Microimager 1000 sector scanning instrument; Ausonics Pty Ltd, Sydney, Australia) and 16 ewes were detected as twin-pregnant. Sheep were offered diets (Table S1) that match their requirements from d 0 to 40 of gestation as stated by NRC [27] and had free access to clean fresh water.

### Experimental design

In vivo experiment, the 16 twin-pregnant Hu were randomly divided into two groups, each consisting of 8 ewes. The control (CON) group received corn oil once a day, whereas the treatment group received a subcutaneous injection of 5 mg/kg/d BPA (purity ≥ 99%, Aldrich Chemical, Milwaukee, WI, USA) that had been mixed sufficiently with corn oil and given once per day (BPA group) from d 40 to 130 of gestation. The dose [28] and time frame [29] of BPA exposure were adopted from previous research on pregnant sheep.

### Tissue collection

Details of tissue collection and processing have been fully described previously [30]. Briefly, on d 130 of gestation, ewes were stunned using a captive-bolt gun (Supercash Mark 2; Acceles and Shelvoke Ltd., Sutton Coldfield, England) followed by exsanguination. The cotyledonary placenta is a distinguishing feature of ruminants, in contrast to the human placenta. The sheep placenta consists of several button-like structures called placentomes that represent contact points between maternal and fetal circulation. The extent of placental differentiation was used as the criterion to classify the sheep placenta in A, B, C and D placentomes types [30]. Placentas were collected, washed, weighed and categorized into their respective types based on previous research [30]. All placentomes were separated and weighed individually and their weights were summed up to determine their total weight. In this study, we used type A placentomes because they represent the early stages of placental differentiation and thus any potential complications upon exposure of the placenta to BPA can be identified. Samples of the fetal side of the placenta, cotyledons (COT), of similar size were obtained from multiple type-A placentomes within 10 cm of umbilical attachment, frozen in liquid nitrogen and preserved under −80 °C, as earlier described [31]. Simultaneously, fetal weights and sex were assessed and recorded at 130 days of gestation for each treatment group; the sex distribution profiles of the fetuses consisted of 10 and 6 males and females for CON ewes and 7 males and 9 females in the BPA ewes, respectively.

### Extraction, purification and culturing of the OTCs

The uteri of pregnant Hu ewes (from d 45 to 60 of gestation) were obtained from a local abattoir and were shipped to our laboratory in a thermal container equipped with a temperature control chamber containing sterile saline kept at 37 °C. The length of the crown to the rump of the developing fetus was used to provide an estimation of the gestational phase [32]. Primary OTCs were harvested from the tissue sections, purified, and cultured using the protocols proposed by Zhang et al. [33]. The extracted primary OTCs were cultured at 37 °C in an incubator with 5% CO_2_ using a complete DMEM/F12 medium consisting of 10% fetal bovine serum (FBS) and 1% penicillin–streptomycin. The OTCs were identified by the immunofluorescence method previously described [33]. Specifically, the cells were incubated for 14 h at 4 °C using 10% normal goat serum-containing rabbit anti-cytokeratin 7 monoclonal antibodies (1:200; Thermo Fisher Scientific). Then, the cells were rinsed, incubated with goat anti-rabbit-Texas Red labelled antibodies (1:100; Thermo Fisher Scientific) for 1 h at 37 °C, mounted with coverslips, stained for 10 min using 0.5 μg/mL 4–6-diamidino-2-phenylindole and finally visualized by the Zeiss Axio Observer microscope (Carl Zeiss, Oberkochen, Germany).

OTCs were introduced into 96-well plates (1 × 10^4^ cells/well) and allowed to develop in a culture mixture consisting of complete DMEM/F12. After overnight incubation, the OTCs were treated with DMEM/F12 complete medium (CON group), 400 μmol/L BPA (BPA group), 400 μmol/L BPA + 0.5 μg/mL tunicamycin (Tm; ER stress activator; Sigma-Aldrich, USA) (BPA + Tm group), or 400 μmol/L BPA + 1 μmol/L 4-phenyl butyric acid (4-PBA; ER stress antagonist; Sigma-Aldrich, USA) (BPA + 4-PBA group) for 24 h with 6 replicates. For this experiment, we first produced BPA within DMSO as the stock solution, thereafter, diluted it to a final concentration of 400 μmol/L using a D/F12 medium. The doses of BPA, Tm, and 4-PBA were adopted from the previous research of Zhang et al. [34] and Wang et al. [35], correspondingly.

Following the 24 h treatment above described, the cells in the 6-well plates were gently rinsed twice using PBS before lysis using RIPA Lysis Buffer R2220 containing 1% PMSF following manufacturer recommendations. The concentration of proteins in the cells was measured spectrophotometrically at 562 nm utilizing a bicinchoninic acid (BCA) assay reagent as recommended by the manufacturer. The extracted proteins were then preserved at −20 °C for further evaluation [36]. All obtained results were normalized to the control, and all calculations were done using the methodology described in an earlier study [37].

### Cell viability

The degree of cell viability was measured using the CCK-8 test as described by the manufacturer. Briefly, OTCs were placed into culture plates with 96 wells (6,000 cells/well) before being treated. The cells were counted and analyzed after 24 h of culture in each of the four different media. All data were normalized to the control value, and computations were done as previously discussed [38].

### Enzyme-linked immunosorbent assay (ELISA)

Type A COT samples and OTCs were analyzed for total antioxidant capacity (T-AOC), superoxide dismutase (SOD), glutathione peroxidase (GSH-Px), and malondialdehyde (MDA) using the respective test kits (Nanjing Jiancheng Biotechnology Institute, China) and following the previously described methodology [39]. Measurements of progesterone (P4) and estrogen (E2) concentrations in OTCs and placental supernatant were performed using ELISA (Cayman Chemical Company, Ann Arbor, MI, USA) in line with specific protocols [40]. Each assay was replicated 6 times.

### Extraction of mitochondria and determination of reactive oxygen species (ROS)

Mitochondria were isolated from either cultivated cells or type A COT using a mitochondrial isolation kit (TransGen Biotech, Beijing, China) and following a published protocol [41]. ROS was determined by incubating mitochondrial precipitate for 15 min at 37 °C with 10 μmol/L 2′7′-dichlorohydrofluorescein, followed by measuring the fluorescence intensity on a microplate reader [42].

### Measurements of mitochondrial membrane potential (ΔΨm) along with adenosine triphosphate (ATP) contents

ΔΨm was derived with the use of the assay kit (Beyotime Institute of Biotechnology in Shanghai, China), and the difference (variation) in ΔΨm was analyzed as per fluorescence emission type observed in the microplate reader (FLx800, Bio-Tek Instruments, Inc., Winnoski, VT, USA). In the presence of a high ΔΨm, JC-1 monomers can form aggregates in the mitochondrial matrix, which fluoresce red (590 nm). In the case of a low ΔΨm value, JC-1 monomers that emit green fluorescence (529 nm) cannot aggregate [43].

Mitochondrial ATP contents were recorded using ATP assay kits (Beyotime Institute of Biotechnology, Shanghai, China) and following a published protocol [44]. Statistical results were reported as fold changes relative to the CON values.

### Mitochondrial complexes I-IV Activities

Mitochondria were ultrasonicated and lysed on ice with the Ultrasonic Processor (Branson, MO, USA) with a 200-W power setting for 3 s, and the procedure was carried out 30 times every 10 s. Next, the collected lysate was centrifuged for 15 min at a rate of 12,000 × *g* and 4 °C. Protein levels were determined using the BCA protein kit (Nanjing Jiancheng Institute of Bioengineering, Jiangsu, China). Subsequently, NADH ubiquinone reductase (complex I), succinate ubiquinone reductase (complex II), ubiquinol cytochrome-c reductase (complex III), and cytochrome-c oxidase (complexes IV) enzymes were assayed using colourimetric kits (Suzhou Comin Biotechnology Co., Ltd., Suzhou, Jiangsu, China). The concentration of total protein was used to standardize the data in each sample [45].

### Cytokine analysis

Using the BioTek synergy HT microplate reader (Bio-Tek Instruments, Winooski, VT, USA), the TNF-α (R&D Systems, Oxford, UK), IL-1β, and IL-6 (BioSource/MED Probe, Camarillo, CA, USA) contents were measured using commercially available kits at 450 nm. The minimum detectable concentrations of the above three factors were 7.0, 10.0 and 35.0 pg/mL, respectively. The coefficients of variation for both the inter- and intra-assays were ≤ 10%. The results are expressed as pg/mL for OTCs and ng/g protein for type A COT tissue, respectively.

### Evaluation of the placental barrier function

The barrier function of type A COT samples was evaluated using the Ussing chamber technology. After being sliced up, the COT samples were placed on the VCC MC6 EasyMount Ussing chamber system (Physiologic Instruments, San Diego, CA, USA). After achieving balance in the Ussing chamber for 15 min, we proceeded to assess the transepithelial electrical resistance (TER) at regular intervals of 15 min for an hour. Thereafter, the paracellular dextran flux (FD4) level was analyzed with the microplate reader (FLx800; Bio-Tek Instruments, Inc., Winooski, VT, USA) [46].

### Quantitative RT-PCR assay

Total RNA from OTCs and type A COT was extracted using TRIzol Reagent (Thermo Fisher, Waltham, MA, USA). Using the reverse transcription kit (Yeasen, Shanghai, China), total RNA (about 5 μg) was applied to produce cDNA. The thermal cycling conditions below were applied for the reverse transcription: 45 min under 25 °C, 30 min under 42 °C, as well as 5 min under 85 °C. Meanwhile, we used Light CycLer®96 Real-Time PCR System (Roche, Switzerland) to conduct RT-PCR, and the PCR protocol was as follows: 5 min denaturation at 95 °C, 10 s amplification under 95 °C, 20 s annealing under 56 °C, along with 20 s extending under 72 °C. A qPCR reaction was set up using SYBR Premix EX Taq (Yeasen, Shanghai, China). The reference gene utilized was β-actin. Table S2 displays the primer sequences that were generated by the NCBI primer design tool. Each well was sampled thrice. To achieve relative quantification, the Ct (2^−ΔΔCt^) value approach was adopted.

### Western blot analysis

The RIPA lysis solution, which included a protease inhibitor (MCE, Monmouth Junction, NJ, USA), was used to prepare the proteins found in the tissues and cells. Whole tissue and cellular protein extracts were first subjected to boiling, then were electrophoretically isolated in 8%–15% SDS-PAGE, followed by deposition on PVDF membranes. After that, the membranes were heated to 25 °C for one and a half hours while being submerged in a TBST solution that included 5% skim milk powder. Primary antibody (1:1,000) was introduced into 5% skim milk, and the membranes were allowed to incubate in the solution at 25 °C for 1.5–2 h. The internal reference for the experiment was the primary antibody against voltage-dependent anion channel (VDAC) or β-actin. Information on antibodies was shown in Table S3. Membranes were rinsed and thereafter incubated using suitable secondary antibodies (1:10,000). Using a digital camera, the signals of immunoreactive proteins were detected. Image-Pro Plus [47] was used for the quantitative analysis of the protein immunoblots.

### Statistical analysis

All data are presented as a mean ± SEM and all statical evaluations were performed by SPSS 16.0 using ANOVA for multiple comparisons and Student's *t*-test for comparing groups. Initially, fetal gender was included in the model, however, it was excluded after the detection of its insignificant effects on the results and the focus was on maternal interventions instead. According to the results of the homogeneity of variance test, Bonferroni or Tamhane T2 analysis was utilized to detect the variations between the two groups. The significance criterion was *P* ≤ 0.05.

## Results

### Fetal weight, placental efficiency, and placental weight, together with the E2 and P4 levels in the type A COT tissues 

**Fig. 1:**
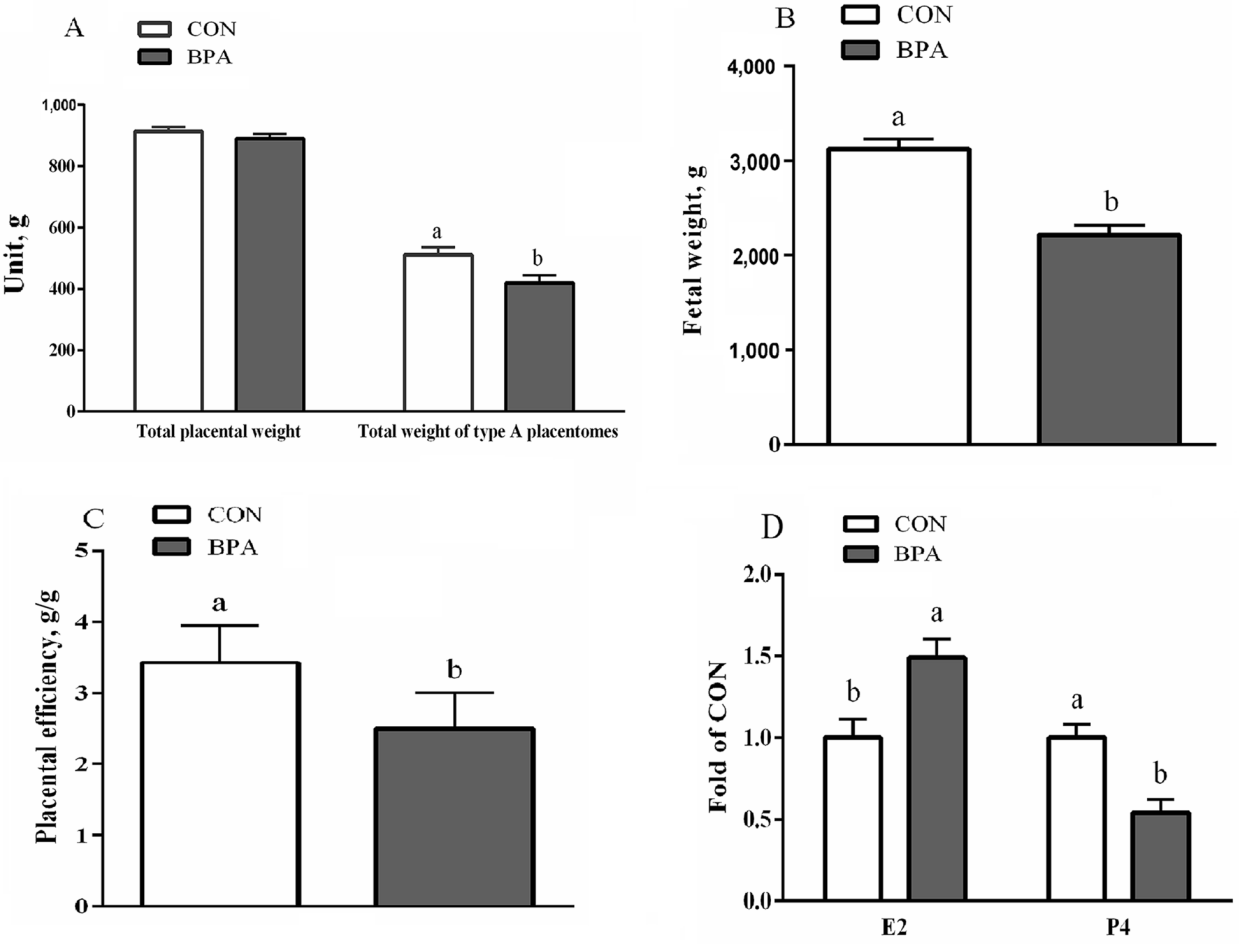
Influence of BPA on the placental efficiency and the levels of E2 and P4 as well as the weights of placentas and fetuses in the type A COT tissues of Hu ewes on d 130 of gestation. **A** Total weights of the placenta and type A placentomes. **B** Weight of the fetuses. **C** Placental efficiency. **D** The levels of E2 and P4. Data are presented as means ± SEM, *n* = 8. Means with no common letter are different (*P* < 0.05). BPA group, pregnant Hu ewes receiving Bisphenol A (5 mg/kg/d mixed sufficiently into corn oil) through subcutaneous injection; CON group, pregnant Hu ewes receiving corn oil only; COT, cotyledonary; E2, estradiol; P4, progesterone

In contrast with the CON ewes, the BPA ewes had substantially lower values for fetal weight, placental efficiency, the total weight of type A placentomes, as well as P4 level; however, the E2 level exhibited higher values (*P* < 0.05) (Fig. 1).

### The activities of mitochondrial complexes, mitochondrial ROS synthesis, ΔΨm, and ATP levels within the type A COT tissues 

**Fig. 2:**
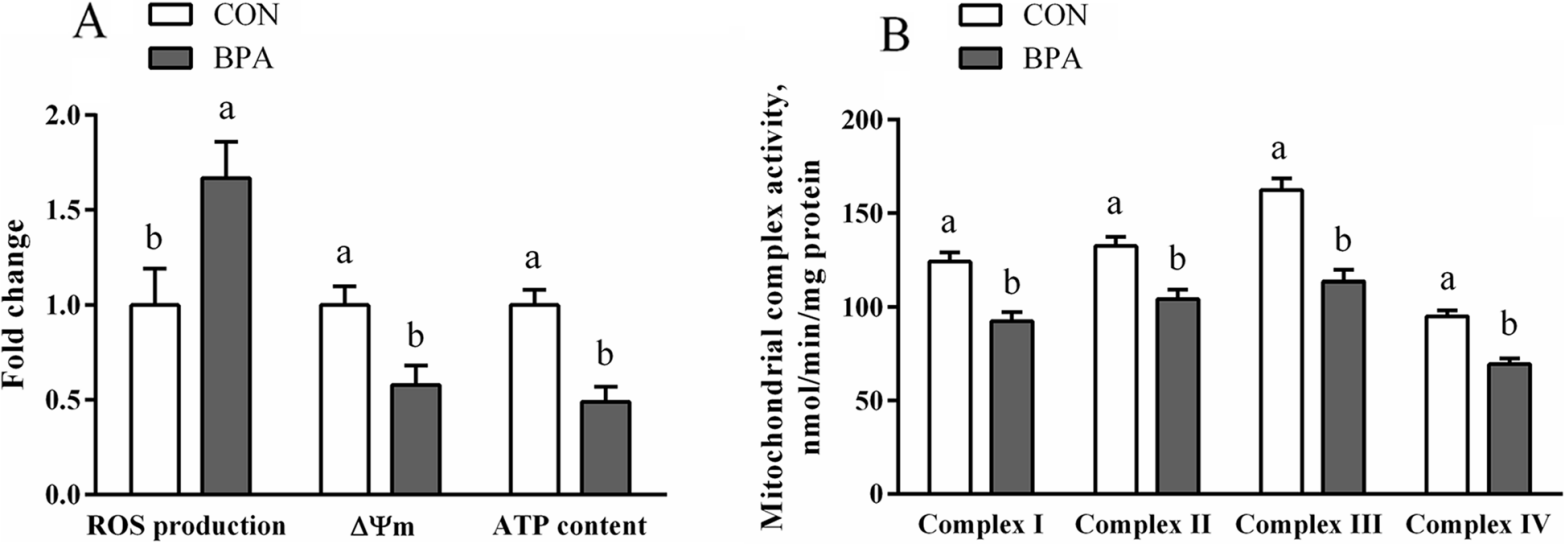
Impact of bisphenol A treatment on mitochondrial ROS levels, ΔΨm, mitochondrial complex activities, together with ATP levels within the type A COT tissues of Hu ewes on d 130 of gestation. **A** The generation of ROS, ΔΨm, and content of ATP. **B** The activity of the mitochondrial complex. Data are presented as means ± SEM, *n* = 8. Means with no common letter are different (*P* < 0.05). ATP, adenosine triphosphate; BPA group, pregnant Hu ewes receiving Bisphenol A (5 mg/kg/d mixed sufficiently into corn oil) through subcutaneous injection; CON group, pregnant Hu ewes receiving corn oil only; COT, cotyledonary; ΔΨm, mitochondrial membrane potential change; ROS, reactive oxygen species

The ROS generation was elevated (*P* < 0.05), whereas the ATP level, ΔΨm and the complexes I-IV activities of mitochondrion were lowered (*P* < 0.05) in type A COT of CON ewes compared to ewes exposed to gestational BPA (Fig. 2).

### Mitochondrial ROS generation, ΔΨm, mitochondria complex activities, together with ATP levels within ovine trophoblast

**Fig. 3:**
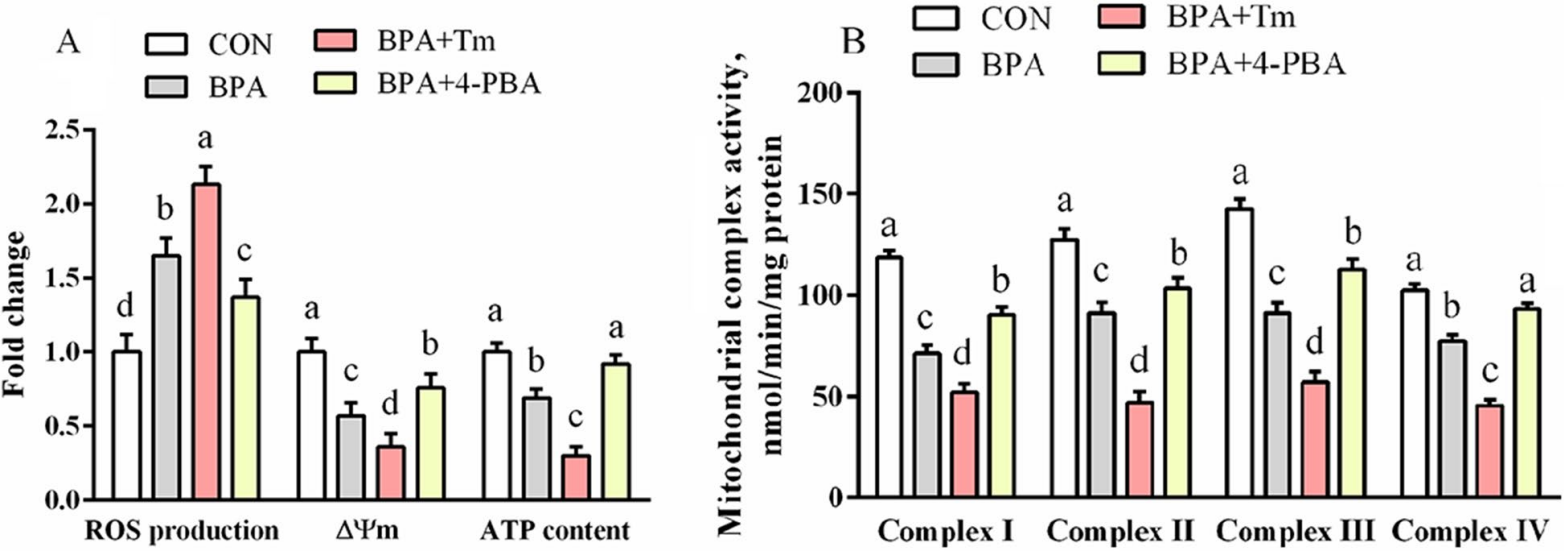
Changes in mitochondrial ROS generation, ΔΨm, the activity of the mitochondrial complex, along with ATP levels within OTCs after exposure to BPA, Tm, and 4-PBA for 24 h. **A** The generation of ROS, ΔΨm, and content of ATP. **B** The activity of the mitochondrial complex. Data are presented as means ± SEM, *n* = 6. Means with no common letter are different (*P* < 0.05). ATP, adenosine triphosphate; BPA group, OTCs treated with 400 μmol/L bisphenol A (BPA); BPA + Tm, OTCs treated with 400 μmol/L BPA + 0.5 μg/mL tunicamycin (Tm; ERS activator); BPA + 4-PBA, OTCs treated with 400 μmol/L BPA + 1 μmol/L 4-phenyl butyric acid (4-PBA; ERS antagonist); CON, OTCs treated with DMEM/F12 complete medium; ΔΨm, mitochondrial membrane potential change; OTCs, ovine trophoblast cells; ROS, reactive oxygen species

The ROS generation was elevated (*P* < 0.05), whereas the ATP level, ΔΨm, and the activity levels of mitochondrial complexes I-IV were lowered (*P* < 0.05) in BPA-treated OTCs compared to the CON OTCs (Fig. 3). The ROS synthesis was enhanced (*P* < 0.05), whereas the ATP content, ΔΨm, and mitochondrial complexes I-IV activities were decreased (*P* < 0.05) in OTCs treated with BPA + Tm compared to the OTCs exposed to BPA only. The ROS levels were lowered (*P* < 0.05), whereas ATP content, ΔΨm, and the activity of mitochondrial complexes I-IV were elevated (*P* < 0.05) in the OTCs treated with BPA + 4-PBA compared to the OTCs exposed to BPA only.

### Antioxidant activities, antioxidative gene levels, and apoptosis of the type A COT tissues

**Fig. 4:**
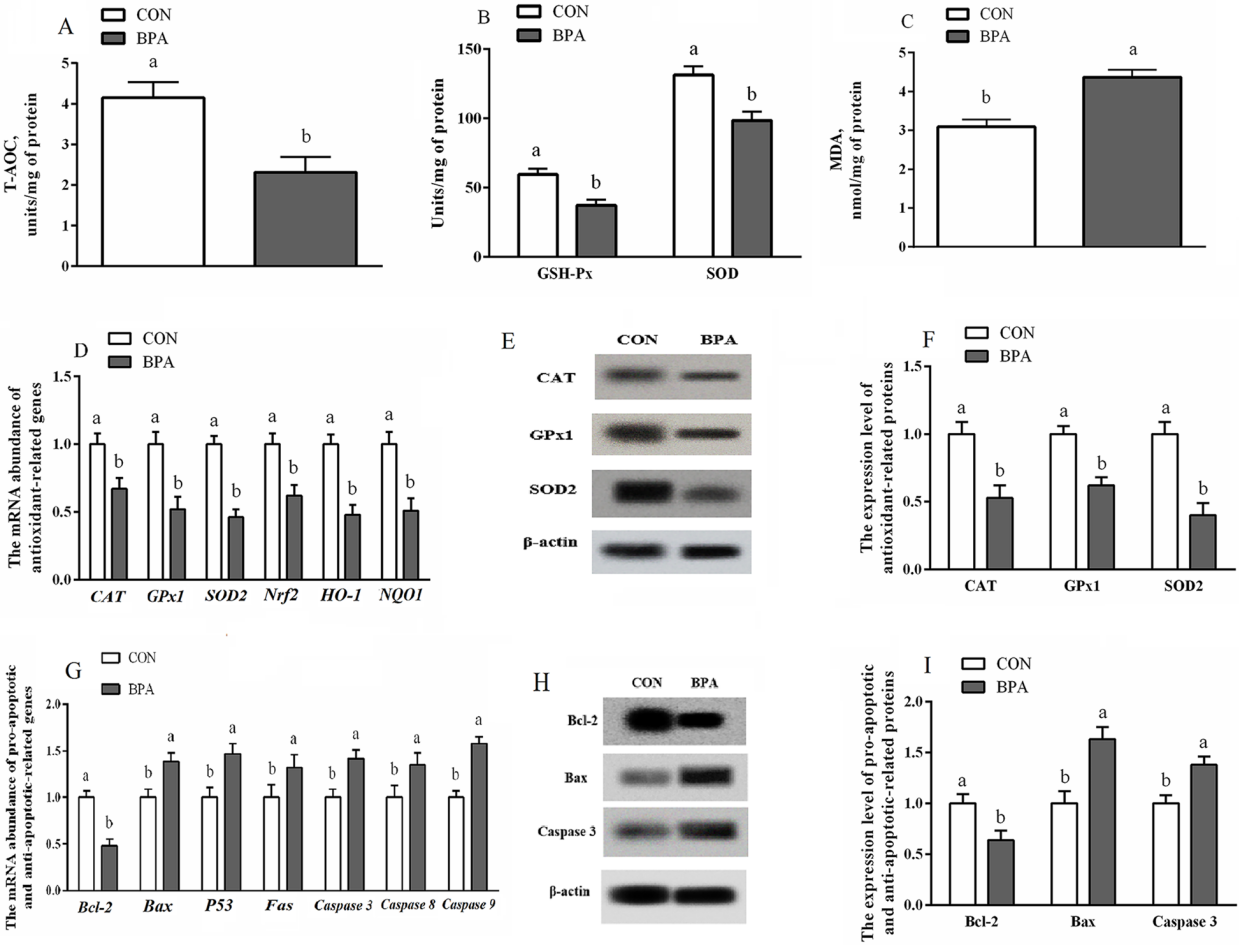
The role of exposure to BPA in antioxidant activities, antioxidation and apoptosis levels, and apoptosis of the type A COT tissues of Hu ewes on d 130 of gestation. **A–C** Antioxidant activity T-AOC (**A**), GSH-Px and SOD (**B**), and MDA (**C**). **D** Antioxidant-associated genes are expressed at the mRNA level. **E** Representative immunoblots showing proteins involved in antioxidant processes. **F** Expressions of antioxidant-related proteins. **G** Apoptotic gene levels. **H** Typical immunoblots of proteins involved in apoptosis. **I **Expressions of proteins that are linked to apoptosis. Data are presented as means ± SEM, *n* = 8. Means with no common letter are different (*P* < 0.05). BPA group, pregnant Hu ewes receiving Bisphenol A (5 mg/kg/d mixed sufficiently into corn oil) through subcutaneous injection; Bax, Bcl-2-associated X protein; Bcl-2, B-cell lymphoma/leukaemia 2; CON group, pregnant Hu ewes receiving corn oil only; CAT, catalase; COT, cotyledonary; Fas, apoptosis antigen 1; GPx1, glutathione peroxidase 1; GSH-Px, glutathione peroxidase; HO-1, heme oxygenase-1; MDA, malondialdehyde; NQO1, quinone oxidoreductase 1; Nrf2, nuclear factor erythroid 2-related factor 2; SOD, superoxide dismutase; T-AOC, total antioxidant capacity

The MDA level was elevated (*P* < 0.05) while the activities of GSH-Px, SOD, and T-AOC were decreased (*P* < 0.05) in type A COT in pregnant ewes exposed to BPA compared to the CON ewes (Fig. 4). The expressions of antioxidant-associated genes (*CAT*, *SOD2*, *GPx1*, *Nrf2*, *NQO1*, and *HO-1*) and proteins (CAT, SOD2, and GPx1), anti-apoptotic related genes and proteins (Bcl2) were decreased (*P* < 0.05), but genes (*Fas*, *Bax*, *P53*, *Caspase 3*, *Caspase 8*, and *Caspase 9*) and proteins (Bax and Caspase 3) related to apoptosis were increased (*P* < 0.05) in type A COT in the BPA ewes compared to the CON ewes.

### The cell viability, the concentration of P4 and E2, antioxidant activities, antioxidative gene levels, and apoptosis of ovine trophoblast

**Fig. 5:**
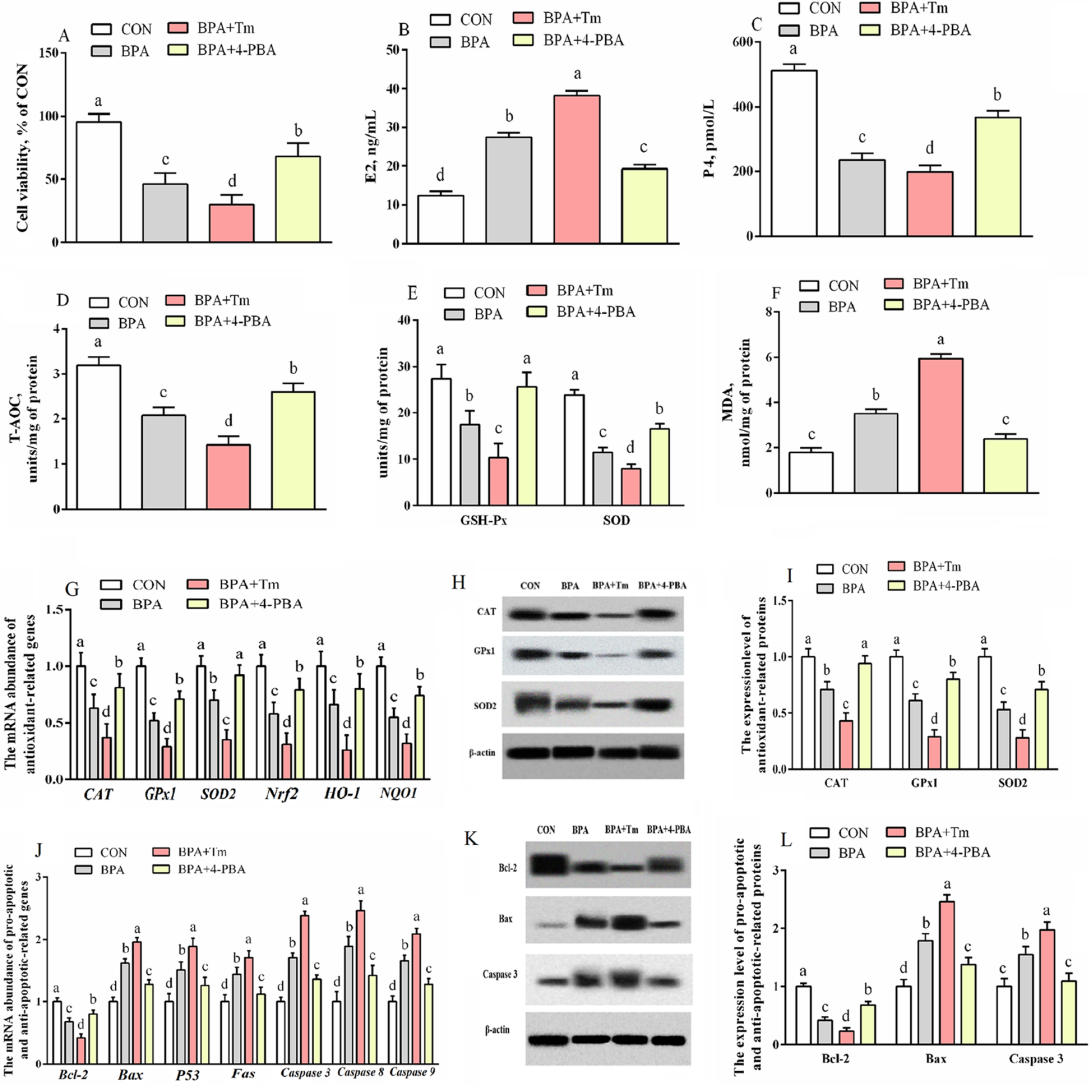
Influence of BPA, Tm, and 4-PBA on the cell viability, concentration of P4 and E2, antioxidant activities, antioxidation, and apoptosis mRNA and protein levels within OTCs at 24 h. **A** Cell viability. **B** E2 level. **C** P4 level. **D–F** Antioxidant activities of T-AOC (**D**), GSH-Px and SOD (**E**), as well as MDA (**F**). **G** Antioxidant-related gene levels. **H** Typical immunoblots for antioxidant-associated proteins. **I** Antioxidant-associated protein expression. **J** Apoptosis-associated gene levels. **K** Typical immunoblots for apoptosis-associated proteins. **L** Apoptosis-associated protein expression. Data are presented as means ± SEM, *n* = 6. Means with no common letter are different (*P* < 0.05). BPA group, OTCs treated with 400 μmol/L bisphenol A (BPA); BPA + Tm, OTCs treated with 400 μmol/L BPA + 0.5 μg/mL tunicamycin (Tm; ERS activator); BPA + 4-PBA, OTCs treated with 400 μmol/L BPA + 1 μmol/L 4-phenyl butyric acid (4-PBA; ERS antagonist); Bax, Bcl-2-associated X protein; Bcl-2, B-cell lymphoma/leukemia 2; Bcl-2, B-cell lymphoma/leukemia 2; Bax, Bcl-2-associated X protein; CON, OTCs treated with DMEM/F12 complete medium; CAT, catalase; E2, estradiol; Fas, apoptosis antigen 1; GSH-Px, glutathione peroxidase; GPx1, glutathione peroxidase 1; HO-1, heme oxygenase-1; MDA, malondialdehyde; NQO1, quinone oxidoreductase 1; Nrf2, nuclear factor erythroid 2-related factor 2; OTCs, ovine trophoblast cells; P4, progesterone; SOD, superoxide dismutase; T-AOC, total antioxidant capacity

BPA exposure significantly elevated (*P* < 0.05) E2 and MDA levels, while reducing (*P* < 0.05) the cell viability, P4 level, and T-AOC, SOD and GSH-Px activities compared to those of the untreated OTCs (Fig. 5). The E2 level and MDA activity were elevated (*P* < 0.05) but cell viability, P4 level, and SOD, GSH-Px, and T-AOC activities were reduced (*P* < 0.05) in OTCs treated with BPA + Tm compared to those in the OTCs exposed to BPA only. The E2 level and MDA activity were decreased (*P* < 0.05) but cell viability, P4 level, and SOD, T-AOC, and GSH-Px activities were up-regulated (*P* < 0.05) in the OTCs treated with BPA + 4-PBA relative to OTCs exposed to BPA only.

The expressions of antioxidant-associated genes (*NQO1*, *GPx1*, *CAT*, *SOD2*, *Nrf2*, and *HO-1*) and proteins (CAT, GPx1, and SOD2), anti-apoptotic related genes and proteins (Bcl2) were reduced (*P* < 0.05), but genes (*P53*, *Fas*, *Bax*, *Caspase 3*, *Caspase 8*, and *Caspase 9*), as well as proteins (Bax and Caspase 3) related to apoptosis, were increased (*P* < 0.05) within BPA-exposed OTCs compared to those in CON OTCs. The levels of antioxidative genes (*GPx1*, *CAT*, *Nrf2*, *SOD2*, *NQO1,* and *HO-1*) and proteins (SOD2, CAT, and GPx1), anti-apoptotic related genes and proteins (Bcl2) were decreased (*P* < 0.05), but genes (*P53*, *Fas*, *Bax*, *Caspase 3*, *Caspase 8*, and *Caspase 9*) together with proteins (Bax and Caspase 3) promoting apoptosis were increased (*P* < 0.05) in the OTCs treated with BPA + Tm relative to those of the BPA-exposed OTCs. The antioxidative genes (*GPx1*, *CAT*, *Nrf2*, *SOD2*, *NQO1,* and *HO-1*) and proteins (SOD2, CAT, and GPx1), anti-apoptotic related genes and proteins (Bcl2) were increased (*P* < 0.05), but genes (*P53*, *Fas*, *Bax*, *Caspase 3*, *Caspase 8* and *Caspase 9*) along with proteins (Bax and Caspase 3) promoting apoptosis were lowered (*P* < 0.05) in the OTCs treated with BPA + 4-PBA compared to those of the BPA-treated OTCs.

###  The inflammation and barrier function in the type A COT tissues

**Fig. 6:**
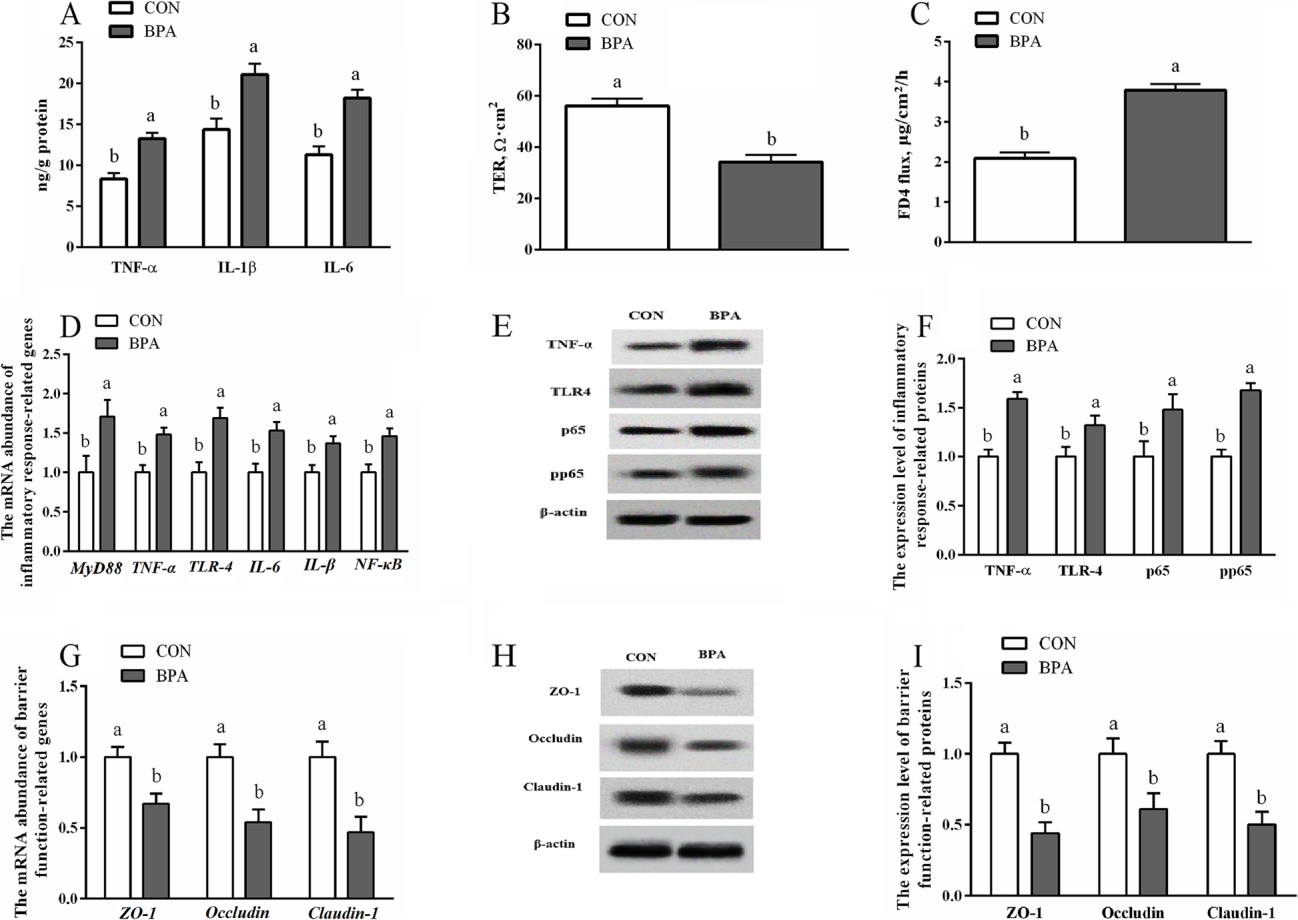
Role of BPA exposure in the inflammatory response and barrier function in the type A COT tissues within Hu ewes on d 130 of gestation. **A** Concentrations of factors (TNF-α, IL-1β and IL-6). **B** TER. **C** FD4. **D** Inflammatory response-related gene levels. **E** Typical immunoblots showing inflammatory response-related proteins. **F** Inflammatory response-related protein levels. **G** Barrier function-related gene levels. **H** Typical immunoblots showing barrier function-related proteins. **I** Barrier function-related protein levels. Data are presented as means ± SEM, *n* = 8. Means with no common letter are different (*P* < 0.05). BPA group, pregnant Hu ewes receiving Bisphenol A (5 mg/kg/d mixed sufficiently into corn oil) through subcutaneous injection; CON group, pregnant Hu ewes receiving corn oil only; COT, cotyledonary; FD4, paracellular dextran flux; IL, interleukin; MyD88, myeloid differentiation factor 88; NF-κB, nuclear factor kappa-B; TER, transepithelial electrical resistance; TNF, tumour necrosis factor; TLR, toll-like receptor; ZO-1, zonula occludens-1

The gestational BPA exposure significantly increased (*P* < 0.05) cytokine concentrations such as IL-6, IL-1β, TNF-α and FD4 flux, but decreased (*P* < 0.05) the value of TER within type A COT in pregnant Hu ewes compared to those in the CON ewes (Fig. 6). The expression levels of immune function-associated genes (*IL-1β*, *IL-6*, *MyD88*, *NF-κB*, *TLR-4* and *TNF-ɑ*), as well as proteins (TLR-4, p65, TNF-ɑ and p-p65), were higher (*P* < 0.05), but barrier function-related genes and proteins (ZO-1, Occludin and Claudin-1) were lowered (*P* < 0.05) in type A COT in the BPA-exposed ewes compared to those in the CON ewes.

###  The inflammation and barrier function in BPA-treated ovine trophoblast

**Fig. 7:**
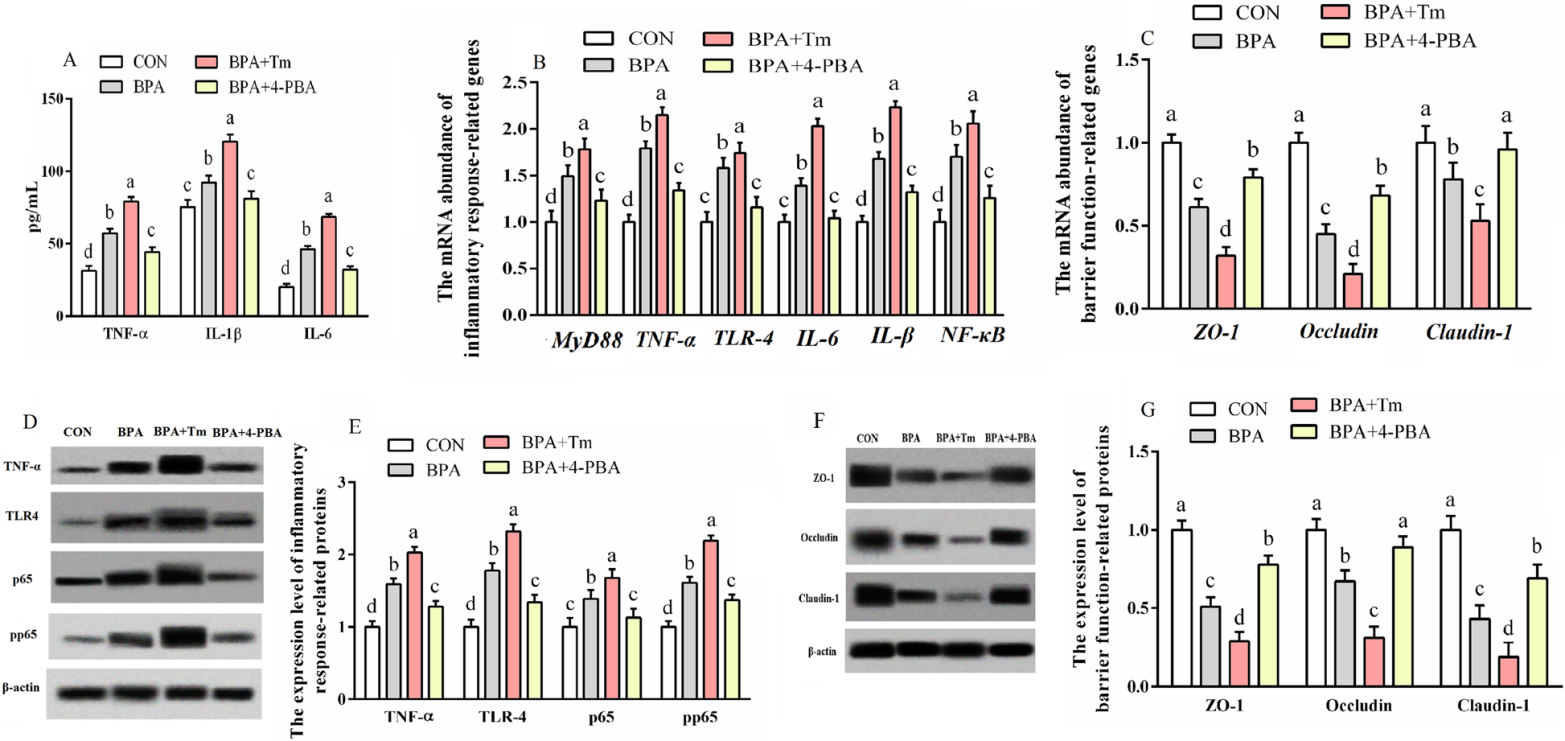
Effects of BPA, Tm and 4-PBA on the inflammatory response and barrier function in OTCs at 24 h. **A** Cytokine concentrations (IL-1β, TNF-α and IL-6). **B** Inflammatory response-related genes. **C** Barrier function-related gene levels. **D** Typical immunoblots showing inflammatory response-related proteins. **E** Inflammatory response-related proteins. **F** Typical immunoblots exhibiting barrier function-related proteins. **G** Barrier function-related protein levels. Data are presented as means ± SEM, *n* = 6. Means with no common letter are different (*P* < 0.05). BPA group, OTCs treated with 400 μmol/L bisphenol A (BPA); BPA + Tm, OTCs treated with 400 μmol/L BPA + 0.5 μg/mL tunicamycin (Tm; ERS activator); BPA + 4-PBA, OTCs treated with 400 μmol/L BPA + 1 μmol/L 4-phenyl butyric acid (4-PBA; ERS antagonist); CON, OTCs treated with DMEM/F12 complete medium; IL, interleukin; MyD88, myeloid differentiation factor 88; NF-κB, nuclear factor kappa-B; OTCs, ovine trophoblast cells; TLR, toll-like receptor; TER, transepithelial electrical resistance; TNF, tumour necrosis factor; ZO-1, zonula occludens-1

The cytokine levels including TNF-α, IL-1β and IL-6, expressions of immune function-associated genes (*IL-1β, TNF-ɑ*, *MyD88*, *IL-6*, *NF-κB* and *TLR-4*) and proteins (p65, TLR-4, TNF-ɑ and p-p65) were greater (*P* < 0.05), but barrier function associated genes and proteins (Claudin-1, Occludin and ZO-1) were lower (*P* < 0.05) in the BPA-treated OTCs compared to those in the untreated OTCs (Fig. 7). The levels of TNF-α, IL-1β and IL-6 cytokines, expressions of immune function-associated genes (*IL-6*, *TNF-ɑ*, *MyD88*, *IL-1β*, *NF-κB* and *TLR-4*) and proteins (p65, TLR-4, TNF-ɑ and p-p65) were higher (*P* < 0.05), but barrier function associated genes and proteins (ZO-1, Occludin and Claudin-1) were lowered (*P* < 0.05) in OTCs treated with BPA + Tm compared to those of the BPA-treated OTCs. The levels of TNF-α, IL-1β and IL-6 cytokines, expressions of immune function-associated genes (*MyD88*, *TNF-ɑ*, *IL-1β*, *IL-6*, *TLR-4* and *NF-κB*) and proteins (p65, TLR-4, TNF-ɑ and p-p65) were lower (*P* < 0.05), but barrier function-associated genes and proteins (ZO-1, Occludin and Claudin-1) were elevated (*P* < 0.05) in OTCs treated with BPA + 4-PBA relative to those of the BPA-exposed OTCs.

### The relative expression of ERS and autophagy mRNA and protein within type A COT tissues 

**Fig. 8:**
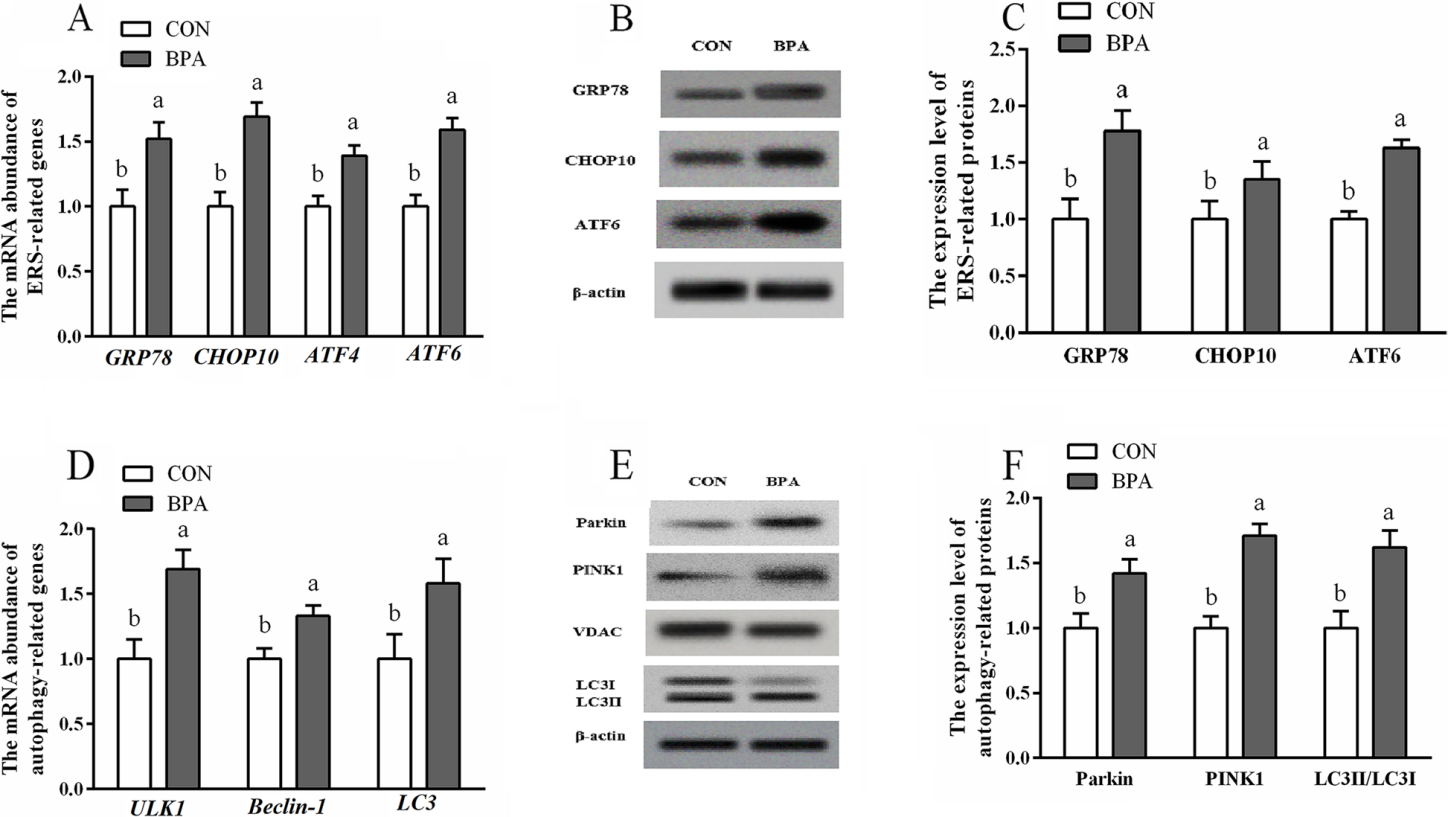
Role of BPA treatment in ERS and autophagy levels of type A COT tissues of Hu ewes on d 130 of gestation. **A** ERS-associated gene levels. **B** Typical immunoblots for ERS-associated proteins. **C** ERS-associated protein levels. **D** Autophagy-associated gene levels. **E** Typical immunoblots showing autophagy-associated proteins. **F** Autophagy-associated protein levels. Data are presented as means ± SEM, *n* = 8. Means with no common letter are different (*P* < 0.05). ATF, activating transcription factor; BPA group, pregnant Hu ewes receiving Bisphenol A (5 mg/kg/d mixed sufficiently into corn oil) through subcutaneous injection; CON group, pregnant Hu ewes receiving corn oil only; COT, cotyledonary; CHOP10, C/EBP homologous protein 10; GRP78, glucose-regulated protein 78; LC3, microtubule-associated protein light chain 3; PINK1, PTEN induced putative kinase 1; ULK1, unc-51 like autophagy activating kinase 1

The expression levels of genes linked to ERS (*ATF6*, *GRP78*, *ATF4* and *CHOP10*) as well as proteins (ATF6, CHOP10 and GRP78) and genes implicated in autophagy (*LC3*, *Beclin-1* and *ULK1*) together with proteins (LC3II/3I, PINK1 and Parkin) were elevated (*P* < 0.05) in the type A COT of the BPA-exposed ewes compared to those of the CON ewes (Fig. 8).

### The relative ERS and autophagy gene and protein levels within BPA-mediated ovine trophoblast

**Fig. 9:**
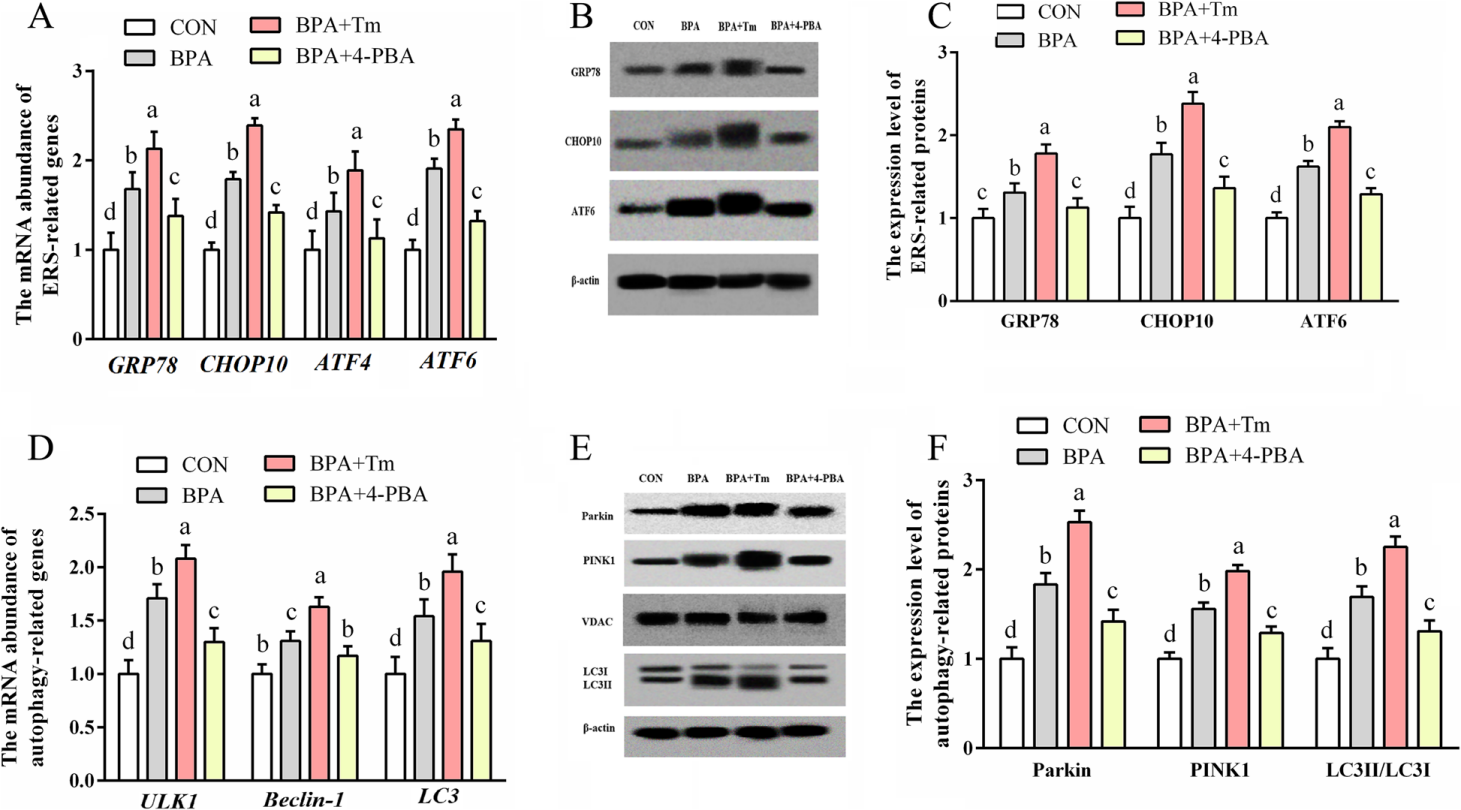
Roles of BPA, Tm, together with 4-PBA in ERS and autophagy levels within OTCs at 24 h. **A** ERS-associated gene levels. **B** Typical immunoblots for ERS-associated proteins. **C** ERS-associated protein levels. **D** Autophagy-associated gene levels. **E** Typical immunoblots showing autophagy-associated proteins. **F** Autophagy-associated protein levels. Data are presented as means ± SEM, *n* = 6. Means with no common letter are different (*P* < 0.05). ATF, activating transcription factor; BPA group, OTCs treated with 400 μmol/L bisphenol A (BPA); BPA + Tm, OTCs treated with 400 μmol/L BPA + 0.5 μg/mL tunicamycin (Tm; ERS activator); BPA + 4-PBA, OTCs treated with 400 μmol/L BPA + 1 μmol/L 4-phenyl butyric acid (4-PBA; ERS antagonist); CON, OTCs treated with DMEM/F12 complete medium; CHOP10, C/EBP homologous protein 10; ERS, endoplasmic reticulum stress; GRP78, glucose-regulated protein 78; LC3, microtubule-associated protein light chain 3; OTCs, ovine trophoblast cells; PINK1, PTEN induced putative kinase 1; ULK1, unc-51 like autophagy activating kinase 1

The levels of ERS-associated genes (*ATF6*, *ATF4*, *CHOP10* and *GRP78*), as well as proteins (ATF6, CHOP10 and GRP78), and autophagy-associated genes (LC3, Beclin-1 and ULK1) and proteins (LC3II/3I, PINK1 and Parkin), increased (*P* < 0.05) in the OTCs exposed to BPA relative to those in CON OTCs (Fig. 9). The ERS-associated genes (*ATF6*, *ATF4*, *CHOP10* and *GRP78*) and proteins (ATF6, CHOP10, ATF4 and GRP78), as well as autophagy-associated genes (*LC3*, *Beclin-1* and *ULK1*) and proteins (LC3II/3I, PINK1 and Parkin) levels were elevated (*P* < 0.05) in the OTCs treated with BPA + Tm compared with those in the BPA-exposed OTCS. The ERS-associated genes (*ATF6*, *ATF4*, *CHOP10* and *GRP78*) and proteins (ATF6, CHOP10 and GRP78), as well as autophagy-associated genes (*LC3*, *Beclin-1* and *ULK1*) and proteins (LC3II/3I, PINK1 and Parkin) levels were decreased (*P* < 0.05) in the OTCs treated with BPA + 4-PBA compared with those in the OTCs exposed to BPA.

## Discussion

Placental efficiency, defined as the fetal birth weight divided by placental mass, is an essential indicator of uterine capacity [48]. In addition, placental efficiency is an indirect measure of the placenta's ability to provide essential nutrients for a developing fetus and it represents the significant factor affecting fetal size [49]. Placental efficiency and fetal size are positively correlated [50]. A birth weight that is less than the reference cut-off, most commonly the 10^th^ percentile, has been considered, for a long time, the primary basis for the FGR [51]. In the previous study, the reduction in placental efficiency and fetal weight occurring at d 65 but not at d 90 of gestation suggests a compensatory mechanism such as an increase in the antioxidants that might overcome the disruptive effects of BPA [29]. In contrast, our results demonstrated that BPA exposure might lead to FGR in pregnant ewes by reducing placental efficiency and fetal weight on d 130 of gestation compared to CON pregnant ewes. These differences might be related in part to breed, litter size, BPA dosage and exposure duration. Furthermore, concerns remain about what these results mean for placental dysfunction due to BPA exposure. Our results indicated that BPA exposure induced placental barrier and endocrine dysfunction, ERS, oxidative stress, and inflammatory responses in the placenta and trophoblasts. In OTCs, the ERS inhibitor (4-PBA) and activator (Tm) were employed to provide clarity on the involvement of ERS in BPA-triggered placental dysfunction in trophoblasts.

Successful pregnancies and healthy fetuses are dependent on the P4 hormone [52]. Previous literature found that mice exposed to noise stress during pregnancy had impaired fetal growth because of the lowered levels of maternal P4 [53]. Similarly, our findings demonstrated that BPA triggered the decrease in P4 levels in maternal placenta and trophoblasts, which ultimately led to FGR in our Hu sheep. Premature delivery occurs when either P4 levels are lowered or the ratio of E2 to P4 is increased, and implantation in the secretory stage is triggered by an increase in P4 and a reduction in E2 [35]. Our findings also suggest that BPA remarkably enhanced E2 production in sheep placenta and trophoblasts, while concurrently eliciting the ERS and decreasing P4 production. We also found that further ERS induction by Tm enhanced the E2 level and reduced the P4 level, whereas ERS inhibition by 4-PBA reduced the E2 level and increased the P4 level in BPA-treated OTCs. Overall, our results indicate that ERS aggravated the BPA-induced placental endocrine dysfunction.

During pregnancy, the placenta serves as an intermediary between the mother and the developing fetus, facilitating the exchange of metabolites, nutrients, and hormones between the two circulatory systems [54]. The placental barrier integrity is of utmost importance for assessing the toxicological effects of BPA on reproduction and fetal development. Therefore, we measured the intercellular tight junctions of the placenta, which are essential for the proper functioning of the placental barrier, to elucidate the role of BPA in adverse pregnancy outcomes [55]. Endothelial proteins found in tight junctions, like claudin-1 and ZO-1, are responsible for regulating the permeation of endothelium monolayers and play a significant role in maintaining the integrity of blood vessels [56]. In the current study, BPA exposure remarkably promoted the FD4 flux but decreased the TER as well as the barrier function-associated genes and proteins (ZO-1, Occludin, Claudin-1) within trophoblasts and sheep placenta, which suggested the presence of placenta barrier dysfunction. We further explored the role of ERS in the regulation of barrier function in OTCs under BPA exposure conditions. We found that the induction of ERS by Tm treatment aggravates the destruction of placental barrier function, and, on the other hand, the inhibition of ERS by 4-PBA alleviated the placental barrier function damage in BPA-treated OTCs. These findings illustrated that the inhibition of ERS can help reduce BPA-induced placenta barrier dysfunction in sheep placenta and trophoblasts.

Oxidative stress (OS) is the constant state of oxidative damage in cells, tissues, or organs generally caused by ROS [57]. Placental ROS has been demonstrated to impact placental development, which could lead to OS and other negative outcomes [58]. There is a strong relationship between placental OS and the biological characteristics of the vasculature and trophoblast [59]. According to our findings, BPA caused a remarkable increase in MDA and ROS levels, as well as a decline in the levels of T-AOC and glutathione, and an attenuation in the antioxidant enzyme activities (CAT and SOD), indicating the occurrence of the placental OS. In cases of intrauterine growth restriction, a vasculature process in the placenta that was similar to atherosclerosis was found. This process is manifested as a smaller diameter of arteries with higher plasma levels of OS indicators [60]. The presence of OS in placental cells may result in FGR by impacting on developing fetus in gestation through the placental vascular system [61]. Therefore, the decreased fetal weight and placental inadequacy that were caused by BPA in our study may be attributed to the presence of OS in the placenta. Previous research has demonstrated that both the OS and the ERS may be the immediate pathways that contribute to apoptosis and cell death [62], and the development of the murine placenta is inhibited when exposed to acrylamide during gestation, which occurs via the suppression of proliferation and the stimulation of apoptosis [63]. According to our findings, treatment with BPA remarkably reduced the count of viable cells in the placenta and induced apoptosis. We discovered that BPA may stimulate the mitochondrial pathway of apoptosis, ultimately resulting in damage to the placenta, as demonstrated by the attenuated Bcl-2 level and the increased caspases-3 and -9 expression. The OS has been shown to cause apoptosis and alter critical apoptotic mediators like Bcl-2 family proteins [64]. Placentas that develop intrauterine growth restriction pathology have elevated levels of apoptosis biomarkers [65]. Consequently, our findings demonstrated that prenatal exposure to BPA impairs placental growth in sheep via activating apoptosis. Furthermore, our study indicated that induction of ERS by Tm aggravates OS and apoptosis, and inhibition of ERS by 4-PBA alleviated OS and apoptosis in BPA-treated OTCs. These findings illustrated that the induction of ERS may aggravate OS and apoptosis in sheep placentas and trophoblasts.

The ER is particularly susceptible to disruptions in the homeostasis of redox conditions [66] and there is a link between ERS and placental inadequacy as well as FRG. Unfolded and misfolded proteins accumulation into ER lumen, which is an outcome of ERS, may be caused by excessive ROS as well as a variety of pathological insults [67]. Large-scale physiological adjustments occur throughout pregnancy, the majority of which are prompted by the placenta, and mitochondria in the placenta are important for both the development of a healthy fetus and the prevention of pregnancy complications [68]. The function of placenta mitochondria and ER has been widely explored, and mitochondria and ER work together to create close-knit functional units in the cell [69]. Chondriosomes are both the most important generator of ROS and their principal target. Increases in mitochondrial ROS and decreases in ΔΨm levels are both indicators of mitochondria malfunction [70] that compromise the efficiency of the chain of electron transport [71]. The decline in ΔΨm is often followed by a reduction in the content of ATP, which signals a degradation of mitochondrial functioning [64]. BPA exposure was shown to disrupt mitochondrial functioning in the placenta, as evidenced by the elevated ROS contents, the reduced ATP generation and ΔΨm, along with the suppressed mitochondrial complex activity in contrast with CON pregnant Hu ewes. Moreover, the induction of ERS increased ROS production and impaired mitochondrial functions, but inhibition of ERS decreased ROS production and improved mitochondrial functions in BPA-treated OTCs. The results proved that the ERS activation may aggravate mitochondrial dysfunctions in sheep placentas and trophoblasts.

Since the placenta represents the bridge between the mother and the developing fetus, and because fetal cells are foreign to the mother's immune system in a way similar to an allograft, maintaining a pregnancy requires delicate maintenance of a balance between immune activation and inhibition in the placenta [72]. Any change in this balance might disturb the normally occurring inflammatory cascade, leading to adverse pregnancy outcomes [73]. The inflammatory process has been linked to the ERS, which is hallmarked by unfolded protein accumulation into ER as well as subsequent unfolded protein response (UPR) signal transduction processes [74]. The UPR also results in the activation of an inflammatory response through the induction of inflammatory cytokines [75]. A previous study demonstrated that ERS has the potential to trigger autophagy [76]. Additionally, autophagy stimulates an upsurge in the synthesis of TNF-α and IL-6. Reduced levels of IL-6 and TNF-α were observed once autophagy was inhibited [77]. It has been reported that intensively activated autophagy over a long time is associated with an excessive cellular inflammatory response [78]. According to our findings, gestational exposure to BPA induced placental ERS, autophagy, and inflammatory response in sheep. Furthermore, the induction of ERS increases autophagy level and inflammatory response, but inhibition of ERS decreases autophagy level and inflammatory response in BPA-treated OTCs. Yet, further research is necessary to confirm how autophagy regulates inflammation and ERS in sheep placental tissue and cells.

## Conclusion

BPA triggers OS, inflammatory responses, autophagy, ERS, and the dysfunction of endocrine and barrier in sheep placenta and trophoblasts. The activation of ERS aggravates BPA-induced OS, inflammatory responses, autophagy, and the dysfunction of endocrine and barrier in OTCs. Conversely, inhibition of ERS decreased BPA-triggered OS, inflammatory responses, autophagy, and the dysfunction of endocrine and barrier in OTCs. Our findings provide unique insights into the mechanisms underlying the involvement of ERS in BPA-triggered placental and fetal development impairments.

### Supplementary Information

**Additional file 1**: **Table S1.** Ingredient and nutrient composition of the experimental diets on a dry matter basis. **Table S2.** Primer sequences used in the real-time PCR. **Table S3.** Details of antibodies used for western blotting.

## Data Availability

All relevant data are either presented within the manuscript or made available online as supplementary materials. The information is accessible upon reasonable request. The study's raw data and associated analyses may be obtained from the corresponding author upon reasonable request.

## Abbreviations

ATF6: Activating transcription factor-6
BCA: Bicinchoninic acid
BPA: Bisphenol A
ER: Endoplasmic reticulum
ERS: ER stress
E2: Estrogen
FBS: Fetal bovine serum
FGR: Fetal growth restriction
GSH-Px: Glutathione peroxidase
MDA: Malondialdehyde
OS: Oxidative stress
OTCs: Ovine trophoblast cells
P4: Progesterone
SOD: Superoxide dismutase
T-AOC: Total antioxidant capacity
TER: Transepithelial electrical resistance
VDAC: Voltage-dependent anion channel
ZO-1: Zonula occludens-1

## References

[1] Wang S, Yang Y, Luo D, Wu D, Liu H, Li M (2020). Lung inflammation induced by exposure to Bisphenol-A is associated with mTOR-mediated autophagy in adolescent mice. Chemosphere.

[2] Mínguez-Alarcón L, Hauser R, Gaskins AJ (2016). Effects of bisphenol A on male and couple reproductive health: a review. Fertil Steril.

[3] Sunman B, Yurdakök K, Kocer-Gumusel B, Özyüncü Ö, Akbıyık F, Balcı A (2019). Prenatal bisphenol a and phthalate exposure are risk factors for male reproductive system development and cord blood sex hormone levels. Reprod Toxicol.

[4] Vićovac L, Jones CJ, Aplin JD (1995). Trophoblast differentiation during formation of anchoring villi in a model of the early human placenta in vitro. Placenta.

[5] Moser G, Windsperger K, Pollheimer J, de Sousa Lopes SC, Huppertz B (2018). Human trophoblast invasion: new and unexpected routes and functions. Histochem Cell Biol.

[6] Rosenfeld CS (2015). Sex-specific placental responses in fetal development. Endocrinology.

[7] Mao J, Jain A, Denslow ND, Nouri MZ, Chen S, Wang T (2020). Bisphenol A and bisphenol S disruptions of the mouse placenta and potential effects on the placenta-brain axis. Proc Natl Acad Sci USA.

[8] Rochester JR (2013). Bisphenol A and human health: a review of the literature. Reprod Toxicol.

[9] Vom Saal FS, Akingbemi BT, Belcher SM, Birnbaum LS, Crain DA, Eriksen M (2007). Chapel Hill bisphenol A expert panel consensus statement: integration of mechanisms, effects in animals and potential to impact human health at current levels of exposure. Reprod Toxicol.

[10] Jalal N, Surendranath AR, Pathak JL, Yu S, Chung CY (2018). Bisphenol A (BPA) the mighty and the mutagenic. Toxicol Rep.

[11] Cao Y, Chen Z, Zhang M, Shi L, Qin S, Lv D (2022). Maternal exposure to bisphenol A induces fetal growth restriction via upregulating the expression of estrogen receptors. Chemosphere.

[12] O’Donnell KJ, Meaney MJ. Fetal origins of mental health: the developmental origins of health and disease hypothesis. Am J Psychiatry. 2017;174:319–28. 10.1176/appi.ajp.2016.16020138 27838934

[13] Crispi F, Bijnens B, Figueras F, Bartrons J, Eixarch E, Le Noble F (2010). Fetal growth restriction results in remodeled and less efficient hearts in children. Circulation.

[14] Niu Q, Chen J, Xia T, Li P, Zhou G, Xu C (2018). Excessive ER stress and the resulting autophagic flux dysfunction contribute to fluoride-induced neurotoxicity. Environ Pollut.

[15] Cybulsky AV (2017). Endoplasmic reticulum stress, the unfolded protein response and autophagy in kidney diseases. Nat Rev Nephrol.

[16] Zhang C, Syed TW, Liu R, Yu J (2017). Role of endoplasmic reticulum stress, autophagy, and inflammation in cardiovascular disease. Front Cardiovasc Med.

[17] Hotamisligil GS (2010). Endoplasmic reticulum stress and the inflammatory basis of metabolic disease. Cell.

[18] Yang Y, Pei X, Jin Y, Wang Y, Zhang C (2016). The roles of endoplasmic reticulum stress response in female mammalian reproduction. Cell Tissue Res.

[19] Park HJ, Park SJ, Koo DB, Lee SR, Kong IK, Ryoo JW (2014). Progesterone production is affected by unfolded protein response (UPR) signaling during the luteal phase in mice. Life Sci.

[20] Yung HW, Hemberger M, Watson ED, Senner CE, Jones CP, Kaufman RJ (2012). Endoplasmic reticulum stress disrupts placental morphogenesis: implications for human intrauterine growth restriction. J Pathol.

[21] Guzel E, Arlier S, Guzeloglu-Kayisli O, Tabak MS, Ekiz T, Semerci N (2017). Endoplasmic reticulum stress and homeostasis in reproductive physiology and pathology. Int J Mol Sci.

[22] Gingrich J, Pu Y, Roberts J, Karthikraj R, Kannan K, Ehrhardt R (2018). Gestational bisphenol S impairs placental endocrine function and the fusogenic trophoblast signaling pathway. Arch Toxicol.

[23] Igwebuike UM (2006). Trophoblast cells of ruminant placentas–A minireview. Anim Reprod Sci.

[24] Huang Q, Chen H, Li J, Oliver M, Ma X, Byck D, Gao Y, Jiang SW (2014). Epigenetic and non-epigenetic regulation of syncytin-1 expression in human placenta and cancer tissues. Cell Signal.

[25] Zhang H, Sun L, Wang Z, Deng M, Nie H, Zhang G (2016). N-carbamylglutamate and L-arginine improved maternal and placental development in underfed ewes. Reproduction.

[26] Russel AJF, Doney JM, Gunn RG (1969). Subjective assessment of body fat in live sheep. J Agric Sci.

[27] NRC. Nutrient requirements of small ruminants: sheep, goats, cervids and new world camelids. Washington: National Academy Press; 2007.

[28] Gauderat G, Picard-Hagen N, Toutain PL, Corbel T, Viguié C, Puel S (2016). Bisphenol A glucuronide deconjugation is a determining factor of fetal exposure to bisphenol A. Environ Int.

[29] Song W, Puttabyatappa M, Zeng L, Vazquez D, Pennathur S, Padmanabhan V (2020). Developmental programming: prenatal bisphenol A treatment disrupts mediators of placental function in sheep. Chemosphere.

[30] Vatnick I, Schoknecht PA, Darrigrand R, Bell AW (1991). Growth and metabolism of the placenta after unilateral fetectomy in twin pregnant ewes. J Dev Physiol.

[31] Zhu MJ, Du M, Nijland MJ, Nathanielsz PW, Hess BW, Moss GE (2009). Down-regulation of growth signaling pathways linked to a reduced cotyledonary vascularity in placentomes of over-nourished, obese pregnant ewes. Placenta.

[32] Sivachelvan M, Ali MG, Chibuzo G (1996). Foetal age estimation in sheep and goats. Small Ruminant Res.

[33] Zhang Y, Shi J, Liu S. Establishment and characterization of a telomerase-immortalized sheep trophoblast cell line. Biomed Res Int. 2016;2016:5808575. 10.1155/2016/5808575 PMC4779524 26998488

[34] Zhang Y, Han L, Yang H, Pang J, Li P, Zhang G (2017). Bisphenol A affects cell viability involved in autophagy and apoptosis in goat testis sertoli cell. Environ Toxicol Phar.

[35] Wang X, Lin P, Li Y, Xiang C, Yin Y, Chen Z, et al. *Brucella suis* vaccine strain 2 induces endoplasmic reticulum stress that affects intracellular replication in goat trophoblast cells in vitro. Front Cell Infect Microbiol. 2016;6:19. 10.3389/fcimb.2016.00019 PMC4746994 26904517

[36] Tang X, Liu B, Wang X, Yu Q, Fang R (2018). Epidermal growth factor, through alleviating oxidative stress, protect IPEC-J2 cells from lipopolysaccharides-induced apoptosis. Int J Mol Sci.

[37] Zhao X, Guo Y, Liu H, Gao J, Nie W. *Clostridium butyricum* reduce lipogenesis through bacterial wall components and butyrate. Appl Microbiol Biotechnol. 2014;98:7549–57. 10.1007/s00253-014-5829-x 24878750

[38] Gonzalez A, Santofimia-Castaño P, Rivera-Barreno R, Salido GM (2012). Cinnamtannin B-1, a natural antioxidant that reduces the effects of H_2_O_2_ on CCK-8-evoked responses in mouse pancreatic acinar cells. J Physiol Biochem.

[39] Xu X, Li M, Chen W, Yu H, Yang Y, Hang L (2016). Apigenin attenuates oxidative injury in ARPE-19 cells thorough activation of Nrf2 pathway. Oxid Med Cell Longev.

[40] Zhang S, Sun C, Zhao S, Wang B, Wang H, Zhang J (2020). Exposure to DEHP or its metabolite MEHP promotes progesterone secretion and inhibits proliferation in mouse placenta or JEG-3 cells. Environ Pollut.

[41] Liu HJ, Qin Y, Zhao ZH, Zhang Y, Yang JH, Zhai DH (2020). Lentinan-functionalized selenium nanoparticles target tumor cell mitochondria via TLR4/TRAF3/MFN1 pathway. Theranostics.

[42] Pipatpiboon N, Pratchayasakul W, Chattipakorn N, Chattipakorn SC (2012). PPARγ agonist improves neuronal insulin receptor function in hippocampus and brain mitochondria function in rats with insulin resistance induced by long term high-fat diets. Endocrinology.

[43] Rehfeldt SCH, Laufer S, Goettert MI (2021). A highly selective in vitro JNK3 inhibitor, FMU200, restores mitochondrial membrane potential and reduces oxidative stress and apoptosis in SH-SY5Y cells. Int J Mol Sci.

[44] Fiorillo M, Scatena C, Naccarato AG, Sotgia F, Lisanti MP (2021). Bedaquiline, an FDA-approved drug, inhibits mitochondrial ATP production and metastasis in vivo, by targeting the gamma subunit (ATP5F1C) of the ATP synthase. Cell Death Differ.

[45] Hargreaves I, Mody N, Land J, Heales S (2018). Blood mononuclear cell mitochondrial respiratory chain complex IV activity is decreased in multiple sclerosis patients: effects of β-interferon treatment. J Clin Med.

[46] He L, Yin Y, Li T, Huang R, Xie M, Wu Z (2013). Use of the Ussing chamber technique to study nutrient transport by epithelial tissues. Front Biosci (Landmark Ed).

[47] Zhu HL, Dai LM, Xiong YW, Shi XT, Liu WB, Fu YT (2022). Gestational exposure to environmental cadmium induces placental apoptosis and fetal growth restriction via Parkin-modulated MCL-1 degradation. J Hazard Mater.

[48] Salavati N, Smies M, Ganzevoort W, Charles AK, Erwich JJ, Plösch T (2019). The possible role of placental morphometry in the detection of fetal growth restriction. Front Physiol.

[49] Tanaka K, Yamada K, Matsushima M, Izawa T, Furukawa S, Kobayashi Y (2018). Increased maternal insulin resistance promotes placental growth and decreases placental efficiency in pregnancies with obesity and gestational diabetes mellitus. J Obstet Gynaecol Res.

[50] Krombeen SK, Shankar V, Noorai RE, Saski CA, Sharp JL, Wilson ME (2019). The identification of differentially expressed genes between extremes of placental efficiency in maternal line gilts on day 95 of gestation. BMC Genomics.

[51] Beune IM, Pels A, Gordijn SJ, Ganzevoort W. Temporal variation in definition of fetal growth restriction in the literature. Ultrasound Obstet Gynecol. 2019;53(5):569–70. 10.1002/uog.19189 30079567

[52] Hartwig IR, Pincus MK, Diemert A, Hecher K, Arck PC (2013). Sex-specific effect of first-trimester maternal progesterone on birthweight. Hum Reprod.

[53] Solano ME, Kowal MK, O’Rourke GE, Horst AK, Modest K, Plösch T (2015). Progesterone and HMOX-1 promote fetal growth by CD8^+^ T cell modulation. J Clin Invest.

[54] Kibschull M, Gellhaus A, Winterhager E (2008). Analogous and unique functions of connexins in mouse and human placental development. Placenta.

[55] Liu X, Zhang F, Wang Z, Zhang T, Teng C, Wang Z (2021). Altered gut microbiome accompanying with placenta barrier dysfunction programs pregnant complications in mice caused by graphene oxide. Ecotoxicol Environ Saf.

[56] Ye J, Liu X (2022). Interactions between endoplasmic reticulum stress and extracellular vesicles in multiple diseases. Front Immunol.

[57] Sha H, Zeng H, Zhao J, Jin H (2019). Mangiferin ameliorates gestational diabetes mellitus-induced placental oxidative stress, inflammation and endoplasmic reticulum stress and improves fetal outcomes in mice. Eur J Pharmacol.

[58] Zhao S, Zhong S, Wang F, Wang H, Xu D, Li G (2020). Microcystin-LR exposure decreased the fetal weight of mice by disturbance of placental development and ROS-mediated endoplasmic reticulum stress in the placenta. Environ Pollut.

[59] Jauniaux E, Greenwold N, Hempstock J, Burton GJ (2003). Comparison of ultrasonographic and Doppler mapping of the intervillous circulation in normal and abnormal early pregnancies. Fertil Steril.

[60] Maisonneuve E, Delvin E, Edgard A, Morin L, Dubé J, Boucoiran I (2015). Oxidative conditions prevail in severe IUGR with vascular disease and Doppler anomalies. J Matern Fetal Neonatal Med.

[61] Al-Gubory KH, Fowler PA, Garrel C (2010). The roles of cellular reactive oxygen species, oxidative stress and antioxidants in pregnancy outcomes. Int J Biochem Cell Biol.

[62] Zhao S, Liu Y, Wang F, Xu D, Xie P (2018). N-acetylcysteine protects against microcystin-LR-induced endoplasmic reticulum stress and germ cell apoptosis in zebrafish testes. Chemosphere.

[63] Yu D, Xie X, Qiao B, Ge W, Gong L, Luo D (2019). Gestational exposure to acrylamide inhibits mouse placental development in vivo. J Hazard Mater.

[64] Zhang H, Liu X, Zheng Y, Zha X, Elsabagh M, Zhang Y (2022). Effects of the maternal gut microbiome and gut-placental axis on melatonin efficacy in alleviating cadmium-induced fetal growth restriction. Ecotoxicol Environ Saf.

[65] Cali U, Cavkaytar S, Sirvan L, Danisman N (2013). Placental apoptosis in preeclampsia, intrauterine growth retardation, and HELLP syndrome: an immunohistochemical study with caspase-3 and bcl-2. Clin Exp Obstet Gynecol.

[66] Gansemer ER, McCommis KS, Martino M, King-McAlpin AQ, Potthoff MJ, Finck BN (2020). NADPH and glutathione redox link cycle activity to endoplasmic reticulum homeostasis. iScience.

[67] Luchetti F, Crinelli R, Cesarini E, Canonico B, Guidi L, Zerbinati C (2017). Endothelial cells, endoplasmic reticulum stress and oxysterols. Redox Biol.

[68] Fisher JJ, Bartho LA, Perkins AV, Holland OJ (2020). Placental mitochondria and reactive oxygen species in the physiology and pathophysiology of pregnancy. Clin Exp Pharmacol Physiol.

[69] Burton GJ, Yung HW, Murray AJ (2017). Mitochondrial-endoplasmic reticulum interactions in the trophoblast: stress and senescence. Placenta.

[70] Zhou Y, Zhou L, Ruan Z, Mi S, Jiang M, Li X (2016). Chlorogenic acid ameliorates intestinal mitochondrial injury by increasing antioxidant effects and activity of respiratory complexes. Biosci Biotechnol Biochem.

[71] Indo HP, Davidson M, Yen HC, Suenaga S, Tomita K, Nishii T (2007). Evidence of ROS generation by mitochondria in cells with impaired electron transport chain and mitochondrial DNA damage. Mitochondrion.

[72] Keeling JW, Khong TY (2007). Fetal and neonatal pathology.

[73] Trevisanuto D, Peruzzetto C, Cavallin F, Vedovato S, Cosmi E, Visentin S (2013). Fetal placental inflammation is associated with poor neonatal growth of preterm infants: a case-control study. J Matern Fetal Neonatal Med.

[74] Carloni S, Favrais G, Saliba E, Albertini MC, Chalon S, Longini M (2016). Melatonin modulates neonatal brain inflammation through endoplasmic reticulum stress, autophagy, and miR-34a/silent information regulator 1 pathway. J Pineal Res.

[75] Zhang K, Kaufman RJ (2008). From endoplasmic-reticulum stress to the inflammatory response. Nature.

[76] Shi M, Sekulovski N, MacLean JA, Hayashi K (2017). Effects of bisphenol A analogues on reproductive functions in mice. Reprod Toxicol.

[77] Qian M, Fang X, Wang X (2017). Autophagy and inflammation. Clin Transl Med.

[78] Yang D, Xiao C, Long F, Su Z, Jia W, Qin M (2018). HDAC4 regulates vascular inflammation via activation of autophagy. Cardiovasc Res.

