# Supplementary material for: Mechanisms underlying the role of endoplasmic reticulum stress in the placental injury and fetal growth restriction in an ovine gestation model
Source: J Anim Sci Biotechnol. 2023 Sep 11;14:117. doi: 10.1186/s40104-023-00919-z (PMC10494380; doi:10.1186/s40104-023-00919-z)
Supplement: Supplementary file 1 — Additional file 1: Table S1. Ingredient and nutrient composition of the experimental diets on a dry matter basis. Table S2. Primer sequences used in the real-time PCR. Table S3. Details of antibodies used for western blotting. [file 40104_2023_919_MOESM1_ESM.docx]

**Table S 1** Ingredient and nutrient composition of the experimental diets on a dry matter basis

| **Items^1^** | **Diet 1**  **(0 to 90 d of gestation)** | **Diet 2**  **(91 to130 d of gestation)** |
| --- | --- | --- |
| Ingredient, % |  |  |
| Chinese wild rye | 50.00 | 45.00 |
| Corn | 35.12 | 31.32 |
| Soybean meal | 12.00 | 20.00 |
| Dicalcium phosphate | 1.67 | 2.34 |
| Calcium carbonate | 0.41 | 0.54 |
| Salt | 0.50 | 0.50 |
| Mineral/vitamin premix^2^ | 0.30 | 0.30 |
| Total | 100 | 100 |
| Nutrient composition^3^ |  |  |
| GE, MJ/kg | 17.63 | 18.49 |
| ME, MJ/kg | 9.23 | 10.03 |
| CP, % | 9.98 | 13.59 |
| MP, % | 6.42 | 8.69 |
| EE, % | 4.21 | 4.59 |
| NDF, % | 37.12 | 32.57 |
| ADF, % | 20.98 | 18.93 |
| Ca, % | 0.57 | 0.81 |
| P, % | 0.45 | 0.69 |

^1^ADF, acid detergent fibre; CP, crude protein; EE, ether extract; GE, gross energy; ME, metabolizable energy; MP, Metabolizable protein; NDF, neutral detergent fibre

^2^The premix provided the following nutrients per kilogram of diet: 30,000 IU vitamin A, 10,000 IU vitamin D, 100 mg vitamin E, 90 mg Fe, 12.5 mg Cu, 50 mg Mn, 100 mg Zn, 0.3 mg Se, 0.8 mg I and 0.5 mg Co

^3^Nutrient levels are analyzed values. The values of ME and MP were estimated according to NRC [27]. Methods for chemical analysis of the diet have been reported by previous study [25]

**Table S2** Primer sequences used in the real-time PCR

| **Gene** | **Sequence (5’→3’)** | **GenBank accession number** |
| --- | --- | --- |
| ERS related genes  *CHOP10*  *GRP78*  *ATF4*  *ATF6* | F: AGGACCACCAGAGGTCACAC  R: TGCCACTTTCCTTTCGTTTT  F: TGAAACTGTGGGAGGTGTCA  R: TCGAAAGTTCCCAGAAGGTG  F: CGAGGTGTTTGTGGGGGACT  R: AGGAGCCTGCCTTAGCCTTG  F: AACCAGTCCTTGCTGTTGCT  R: CTTCTTCTTGCGGGACTGAC | AY943948.1  DQ029323.1  GAAI01000637.1  AY942654 |
| Autophagy related genes |  |  |
| *ULK1* | F: CAACCACAAAGACCGCATGG  R: CCCTGAGCTCGGGTATGAG | XM_015101564.1 |
| *Beclin-1* | F: CGGACCGGAAAGTAGCTGAAG  r: GCTGTGGCAAGTAATGGAGC | XM_004012945.3 |
| *LC3* | f: TGTCAACATGAGCGAGTTGGT  r: GCTCGTAGATGTCCGCGATG | XM_012114930.2 |
| Antioxidant-related genes |  |  |
| *CAT* | F：CCATCTGAAAGACGCACAGC | GQ421282.1 |
|  | r：ATGCGGGAGCCATACTCAG |  |
| *GPx1* | F：GCAACCAGTTTGGGCATCAG | JF728302.1 |
|  | r：GCCATTCACCTCGCACTTTT |  |
| *SOD2* | F: TCACAGCATCTTCTGGACAA | NM_001280703 |
|  | R: TGCTCCTTATTGAAGCCAAG |  |
| *Nrf2*  *HO-1*  *NQO1* | F: CGAGCCGGTGTGAGTAGA  R: TTCCGTGGCCCAGTGTAAAG  F: TGGAGGAGGAGATAGAACGC  R: CCTGGAGTCGCTGAACATAG  F: TTCTGTGGCTTCCAGGTCTT  R: TCCAGACGTTTCTTCCATCC | AY369137.1  XM_015094843.1  XM_004015102.3 |
| Pro-apoptotic and anti-apoptotic genes  *Bax*  *Bcl-2* | F: ATGGGCTGGACATTGGACTT  R: ACTGTCTGCCATGTGGGTGT  F: CGAGTGGCGGCTGAAAT  R: GGTCTGCCATGTGGGTGTC | AF163774.1  HM630309.1 |
| *P53* | F: TTCCCCTTCCCTCAACAAGC  R: GCGCGTAAATTCCCTTCCAC | NM_001009403.1 |
| *Fas* | F: TTTTGCTGTCAGCCTTGTCC  r: TGTTCCACTTCTAGCCCATG | NM_001123003.1 |
| *Caspase 3* | F: TCAGGGAAACCTTCACGAGC  R: CCTCGGCAGGCCTGAATAAT | XM_027962551.1 |
| *Caspase 8* | F: TGAAGGTTCCAGGATTCGCC  R: GGCTTAGGAACTTGAGGGCA | XM_004004822.1 |
| *Caspase 9* | F: GCCAAGCCAAGGAAAACTCG  R: CACGGCAGAAGTTCACGTTG | XM_042257438.1 |
| Inflammatory response related genes  *MyD88*  *TNF-α*  *TLR-4*  *IL-6*  *IL-1β*  *NF-κB*  Barrier function related genes  *ZO-1*  *Occludin*  *Claudin-1* | F: ATGGTGGTGGTTGTCTCTGAC  R: GGAACTCTTTCTTCATTGGCTTGT  f: ACACCATGAGCACCAAAAGC  r: AGGCACAAGCAACTTCTGGA  F: GGCATCATCTTCATCGTCCT  R: CCACTCCAGGTAGGTGTTCC  F: AGGAAAAAGATGGATGCTTCCA  r: GACCAGCAGTGGTTTTGATCAA  F: CGTCTTCCTGGGACGTTTTAG  R: CTGCGTATGGCTTCTTTAGGG  F: ATTCAGCCCTTTGCCCATCT  R: ATGGGATGTCAGTGGCGTTA  F: CGACCAGATCCTCAGGGTAA  R: AATCACCCACATCGGATTCT  F: GTTCGACCAATGCTCTCTCAG  R: CAGCTCCCATTAAGGTTCCA  F: CACCCTTGGCATGAAGTGTA  R: AGCCAATGAAGAGAGCCTGA | GQ221044.1  NM_001024860.1  NM_001135930.1  NM_001009392  NM_001009465  EF121765.1  XM_015101953.2  XM_015101256.1  NM_001185016.1 |
| *β-actin* | F: GCTCTTCCAGCCGTCCTT  R: TGAAGGTGGTCTCGTGAATGC | NM_001009784.1 |

ATF, activating transcription factor; Bax, Bcl-2 associated X protein; Bcl-2, B-cell lymphoma/leukaemia 2; CAT, catalase; CHOP10, C/EBP homologous protein 10; ERS, endoplasmic reticulum stress; F, forward primer; Fas, apoptosis antigen 1; GRP78, glucose-regulated protein 78; GPx1, glutathione peroxidase 1; HO-1, heme oxygenase-1; LC3, microtubule-associated protein light chain 3; MyD88, myeloid differentiation factor 88; NF-κB, nuclear factor kappa-B; Nrf2, nuclear factor erythroid 2-related factor 2; NQO1, quinone oxidoreductase 1; R, reverser primer; SOD2, superoxide dismutase 2; TNF, tumor necrosis factor; TLR, toll-like receptor; IL, interleukin; ULK1, unc-51 like autophagy activating kinase 1; ZO-1, zonula occludens-1

**Table S3** Details of antibodies used for western blotting

| **Antibodies** | **Cat NO.** | **Source** | **Dilutions of Western blot** |
| --- | --- | --- | --- |
| ERS related antibodies |  |  |  |
| Anti-GRP78 | AF0171 | Beyotime Biotechnology (Shanghai, China) | 1:1,000 |
| Anti-CHOP10 | AF6684 | Beyotime Biotechnology (Shanghai, China) | 1:1,000 |
| Anti-ATF6 | AF6243 | Beyotime Biotechnology (Shanghai, China) | 1:1,000 |
| Autophagy related antibodies |  |  |  |
| Anti-Parkin | AF7680 | Beyotime Biotechnology (Shanghai, China) | 1:1,500 |
| Anti-PINK1 | AF7755 | Beyotime Biotechnology (Shanghai, China) | 1:1,000 |
| Anti-LC3 | AF5225 | Beyotime Biotechnology (Shanghai, China) | 1:1,000 |
| Antioxidant related antibodies |  |  |  |
| Anti-CAT | AF0084 | Beyotime Biotechnology (Shanghai, China) | 1:1,000 |
| Anti-GPx1 | AF7017 | Beyotime Biotechnology (Shanghai, China) | 1:2,000 |
| Anti-SOD2 | NB100-1992 | Novus (CO, USA) | 1:5,000 |
| Pro-apoptotic and  anti-apoptotic antibodies |  |  |  |
| Anti-Caspase 3 | AF835 | Novus (CO, USA) | 1:1,000 |
| Anti-Bcl-2 | AF0060 | Beyotime Biotechnology (Shanghai, China) | 1:1,000 |
| Anti-Bax | AF0057 | Beyotime Biotechnology (Shanghai, China) | 1:1,000 |
| Inflammatory response  related antibodies |  |  |  |
| Anti-TNF-α  Anti-TLR4  Anti-p65 | AF8208 AF8187  AF0246 | Beyotime Biotechnology (Shanghai, China)  Beyotime Biotechnology (Shanghai, China)  Beyotime Biotechnology (Shanghai, China) | 1:1,500  1:1,000  1:1,000 |
| Anti-pp65  Barrier function related antibodies  Anti-ZO-1  Anti-Occludin  Anti-Claudin-1 | AN365  AF8394  bs-10011R bs-10008R | Beyotime Biotechnology (Shanghai, China)  Beyotime Biotechnology (Shanghai, China)  Bioss Biotechnology (Beijing, China)  Bioss Biotechnology (Beijing, China) | 1:500  1:1,000  1:1,000  1:1,000 |
| Anti-β-actin | bs-0061R | Bioss Biotechnology (Beijing, China) | 1:2,000 |
| Anti-VDAC | bs-23683R | Bioss Biotechnology (Beijing, China) | 1:1,000 |
| HRP-labelled goat anti-rabbit IgG (secondary antibodies) | A0208 | Beyotime Biotechnology (Shanghai, China) | 1:1,000 |

ATF, activating transcription factor; Bax, Bcl-2 associated X protein; Bcl-2, B-cell lymphoma/leukaemia 2; CAT, catalase; CHOP10, C/EBP homologous protein 10; ERS, endoplasmic reticulum stress; GRP78, glucose-regulated protein 78; GPx1, glutathione peroxidase 1; HRP, horseradish peroxidase; LC3, microtubule-associated protein light chain 3; p65, nuclear factor kappa B; PINK1, PTEN induced putative kinase 1; SOD2, superoxide dismutase 2; TNF, tumor necrosis factor; TLR, toll-like receptor; VDAC, voltage dependent anion channel; ZO-1, zonula occludens-1
